# The Subcellular Proteome of a Planctomycetes Bacterium Shows That Newly Evolved Proteins Have Distinct Fractionation Patterns

**DOI:** 10.3389/fmicb.2021.643045

**Published:** 2021-05-04

**Authors:** Christian Seeger, Karl Dyrhage, Mayank Mahajan, Anna Odelgard, Sara Bergström Lind, Siv G. E. Andersson

**Affiliations:** ^1^Science for Life Laboratory, Molecular Evolution, Department of Cell and Molecular Biology, Biomedical Centre, Uppsala University, Uppsala, Sweden; ^2^Department of Chemistry-BMC, Analytical Chemistry, Uppsala University, Uppsala, Sweden

**Keywords:** planctomycetes, proteomics, paralogs, protein evolution, serine/threonine kinases

## Abstract

The *Planctomycetes* bacteria have unique cell architectures with heavily invaginated membranes as confirmed by three-dimensional models reconstructed from FIB-SEM images of *Tuwongella immobilis* and *Gemmata obscuriglobus*. The subcellular proteome of *T. immobilis* was examined by differential solubilization followed by LC-MS/MS analysis, which identified 1569 proteins in total. The Tris-soluble fraction contained mostly cytoplasmic proteins, while inner and outer membrane proteins were found in the Triton X-100 and SDS-soluble fractions, respectively. For comparisons, the subcellular proteome of *Escherichia coli* was also examined using the same methodology. A notable difference in the overall fractionation pattern of the two species was a fivefold higher number of predicted cytoplasmic proteins in the SDS-soluble fraction in *T. immobilis*. One category of such proteins is represented by innovations in the *Planctomycetes* lineage, including unique sets of serine/threonine kinases and extracytoplasmic sigma factors with WD40 repeat domains for which no homologs are present in *E. coli.* Other such proteins are members of recently expanded protein families in which the newly evolved paralog with a new domain structure is recovered from the SDS-soluble fraction, while other paralogs may have similar domain structures and fractionation patterns as the single homolog in *E. coli*. The expanded protein families in *T. immobilis* include enzymes involved in replication-repair processes as well as in rRNA and tRNA modification and degradation. These results show that paralogization and domain shuffling have yielded new proteins with distinct fractionation characteristics. Understanding the molecular intricacies of these adaptive changes might aid in the development of a model for the evolution of cellular complexity.

## Introduction

Members of the *Planctomycetes* have been classified as bacteria by phylogenetic inferences based on both rRNA gene and concatenated protein sequences, as reviewed in Wiegand et al. (2018). Despite their classification as prokaryotes, they carry many fascinating traits that set them apart from other bacteria, most strikingly an elaborate intracellular membrane system, as shown for example in electron tomography studies of *Gemmata obscuriglobus* (Santarella-Mellwig et al., 2013; Sagulenko et al., 2014) and cryo electron tomography studies of *Planctopirus limnophila* (Boedeker et al., 2017) and *Tuwongella immobilis* (Mahajan et al., 2020a). Also unlike typical bacteria, an early diverging lineage in the phylum called the anammox bacteria contain a membrane-bounded organelle inside the cytoplasm, which is involved in anaerobic ammonium oxidation (Neumann et al., 2014).

In addition, it has been suggested that a few species, such as *G. obscuriglobus*, contain a nuclear body compartment (Fuerst and Webb, 1991; Lindsay et al., 2001; Lee et al., 2009; Sagulenko et al., 2014), a claim which is, however, not accepted by most other researchers in the field (Wiegand et al., 2018; Jogler et al., 2019). In a search for evidence for a nuclear membrane in *G. obscuriglobus*, a density gradient fractionation technique was used to purify the membranes and analyze the proteins associated with them (Sagulenko et al., 2017). Three distinct membrane types were identified, one of which was enriched for proteins such as NADH dehydrogenase and ATP synthase, suggesting that it corresponds to the inner cytoplasmic membrane, while another layer was thought to represent the membranes of vesicles from what has been referred as the paryphoplasm. The third layer contained visible pores on the surfaces of the membranes that were interpreted as nuclear pore-like structures, and it was hypothesized that this layer corresponds to a membrane that surrounds the chromosome (Sagulenko et al., 2017).

The evolutionary implications of these intriguing eukaryotic-like traits (membrane invaginations, intracellular organelles and nuclear bodies) have been intensively debated (Forterre and Gribaldo, 2010; Fuerst and Sagulenko, 2011; Mcinerney et al., 2011; Wiegand et al., 2018; Jogler et al., 2019), despite of which no consensus has yet been reached as to whether all of these membrane layers exist and if so, whether they are analogous or homologous to those in eukaryotes.

Moreover, also the cell envelope has unique features that distinguish it from that of other gram-negative bacteria. For example, the cell wall has been suggested to be mainly composed of proteins instead of a peptidoglycan (König et al., 1984; Liesack et al., 1986). Consistently, cysteine-rich proteins with YTV domains have been identified in the cell wall preparations of *Rhodopirellula baltica* (Hieu et al., 2008) and members the *Gemmataceae* (Sagulenko et al., 2017; Mahajan et al., 2020a). Bioinformatics studies have shown that genes for the YTV domain proteins are solely present in the *Planctomycetales*, and these genes have not been detected in early diverging species of the phylum nor in other bacteria (Mahajan et al., 2020a).

Genes for peptidoglycan biosynthesis showed the converse pattern, being present in the early diverging members of the *Planctomycetes* but notably absent in most species of the *Planctomycetales*, including the *Gemmataceae* (van Teeseling et al., 2015; Mahajan et al., 2020a; Wiegand et al., 2020). Furthermore, a strong correlation was observed between the phyletic presence/absence patterns of genes for peptidoglycan biosynthesis and the MreB protein complex involved in cell elongation (Mahajan et al., 2020a). Based on these results, it was suggested that the tight coordination between peptidoglycan biosynthesis, cell elongation and cell division has been lost from many species in the *Planctomycetales*, thereby enabling invaginations of the inner cytoplasmic membrane (Mahajan et al., 2020a).

In this study, we have turned to *T. immobilis*, a closely related species to *G. obscuriglobus* in the *Gemmataceae* family (Kulichevskaya et al., 2017; Seeger et al., 2017) to study the expression of newly evolved proteins that are unique to the family. The genome of this species is only 6.7 Mb, as compared to 9.2 Mb for its sister species *G. obscuriglobus* (Mahajan et al., 2020b). A broad comparative genomics study predicted a massive gain of proteins by duplication and divergence at multiple nodes with the *Gemmataceae* (Mahajan et al., 2020b). The novel protein families were associated with functions such as signal transduction, transcription regulation, replication-repair processes, cell wall biogenesis, secretory pathways and biopolymer transport protein complexes. Many of the recently evolved proteins display unique domain combinations, suggesting that gene duplications and domain shuffling events have been important sources of innovation (Mahajan et al., 2020b).

We have analyzed the expression patterns of proteins predicted to be located in the cytoplasm vs. the inner and outer membranes in *T. immobilis*, using a subcellular fractionation protocol based on differential protein solubility. The results show that several of the recently duplicated and diverged proteins have a different biochemical fractionation pattern than their ancestral homologs, indicative of altered physicochemical properties and/or different subcellular locations. The findings are discussed in relation to previous interpretations of the cell organization of the *Planctomycetes.*

## Materials and Methods

### FIB-SEM

*T. immobilis* and *G. obscuriglobus* were cultivated on M1 agar plates for 4 days at 32°C and stored at room temperature between 1 and 2 days before processing. Bacteria were transferred directly from the plates into frozen hexadecane using a high-pressure freezer, HPM100 (Leica). Frozen samples were freeze substituted in Acetone containing 0.5% Uranyl acetate, 1% OsO_4_ and 5% water with a linear warm up of 5°/h from −90 to 20°C. Samples were washed and infiltrated in Durcupan resin. Samples were mounted on an SEM-pin and coated with 5 nm Pt before FIB-milling. Reconstruction was performed using 3dmod as part of IMOD 4.9 on cells that displayed healthy morphology (full membrane integrity, high contrast in periplasm and cytoplasm). Supplementary Table 1 contains a summary with the number of segmented slices per cell and number of manually segmented contours per cell and cellular structure. Image stacks and corresponding segmentations have been deposited at the EMPIAR archive (Iudin et al., 2016) under the EMPIAR accession codes EMPIAR-10553 (*G. obscuriglobus*) and EMPIAR-10554 (*T. immobilis*).

### Protein Extraction for Proteomics Analyses

*T. immobilis* and *Escherichia coli* were grown in batch culture (*T. immobilis*: 3 × 200 mL M1 medium, 36°C, 180 rpm; *E. coli*: 3 × 200 mL LB medium, 37°C, 160 rpm) until cells reached early stationary phase (*T. immobilis*: 66 h, *E. coli*: 2.5 h). Cells were harvested by centrifugation at 4,500 × g for 10 min at 4°C into 50 mL tubes and washed two additional times in ice-cold 50 mM Tris, pH 7.5, followed by centrifugation at 4,500 × g for 10 min at 4°C. The washed cell pellets were stored at −20°C until protein extraction.

The frozen cell pellets (approximately 500 mg wet weight per pellet) were thawed on ice and re-suspended in 5 mL 50 mM Tris, 10 mM EDTA, 1 × SigmaFast protease inhibitors (Sigma Aldrich), pH 7.5 (*Buffer A*). Cells were lysed on ice by sonication using a Vibra cell sonicator (3 mm probe, *T. immobilis*: 20 × 10 s at 60–70% amplitude, 20 s pause; *E. coli*: 40 × 15 s at 70% amplitude, 30 s pause). Unbroken cells and debris were removed by centrifugation at 10,000 × g for 10 min at 4°C. The supernatant was carefully removed and centrifuged one additional time at 4,500 × g for 20 min at 4°C. Successful removal of unbroken cells and debris was evaluated by phase-contrast microscopy.

The cleared supernatants were centrifuged at 100,000 × g for 40 min at 4°C in a Beckman L7-55 ultracentrifuge (Beckman Coulter) using a Ti90 rotor to pellet the membrane fractions. The supernatants from the replicate samples (S1.1, S1.2, S1.3), containing the Tris-soluble protein fraction were distributed into aliquots and stored at −20°C. The remaining membrane pellets (P1.1, P1.2, P1.3) were rinsed twice in 50 mM Tris, 10 mM MgCl_2_, 1 × protease inhibitors, pH 7.5 (*Buffer B*) before re-suspending the pellets in 3 mL of Buffer B. The washed membranes were centrifuged at 100,000 × g for 40 min at 4°C and the supernatant was discarded.

The washed membrane pellets were rinsed two times in Buffer B, before being re-suspended in 1 mL 50 mM Tris, 10 mM MgCl_2_, 2% (v/v) TX-100, 1 × protease inhibitors, pH 7.5 (*Buffer C*). After shaking for 10 min at room temperature, the pellets were dissolved by gentle sonication on ice, followed by shaking for 30 min at room temperature. The solubilized membranes were centrifuged at 100,000 × g for 40 min at 4°C. The supernatants containing the Tris/TX-100-soluble fraction (S2.1, S2.2, S2.3) were distributed into aliquots and stored at −20°C. The remaining pellets (P2.1, P2.2, P2.3) were re-suspended in Buffer C, gently sonicated and shaken for 30 min at room temperature on an orbital shaker. After ultracentrifugation (100,000 × g, 40 min, 4°C), the supernatants were discarded and the pellets were washed one additional time following the same procedure.

The washed pellets (P2.1, P2.2, P2.3) were re-suspended in 400 μL 50 mM Tris, 10 mM MgCl_2_, 2% (v/v) SDS, pH 7.5, followed by orbital shaking at room temperature for 45 min. The suspension was cleared by centrifugation at 17,000 × g for 30 min at room temperature using a benchtop centrifuge. The supernatants containing the SDS-soluble protein fraction (S3.1, S3.2, S3.3) were transferred into aliquots and stored at −20°C. Prior to in-solution tryptic digestion and LC-MS/MS analysis, TX-100 and SDS were removed from samples S2.1-S2.3 and S3.1-S3.3 by using Detergent Removal Spin Columns (Thermo Fisher Scientific Pierce) by following the manufacturer’s instructions. Spin columns were equilibrated with 50 mM Tris, 8 M urea, pH 7.5.

SDS-PAGE was performed using NuPAGE 4–12% Bis-Tris Protein Gels (Life Technologies). Samples were heated for 10 min at 70°C in 1× NuPAGE Sample Reducing Agent and 1× NuPAGE LDS Sample Buffer. Electrophoresis was performed in 1× NuPAGE MOPS SDS Running Buffer supplemented with NuPAGE Antioxidant at 200 V for 55 min. Novex Sharp Unstained Protein Standard was used a molecular weight marker. Staining was performed with SimplyBlue SafeStain following a microwave procedure according to the instructions of the manufacturer. Images were acquired on a ChemiDoc XRS + Imager (Bio-Rad). Intensity profiles were obtained after background subtraction in ImageJ 1.49v (100 pixel rolling ball radius, separate colors).

### Transmission Electron Microscope and UV-VIS Spectroscopy

To evaluate the different *T. immobilis* subcellular fractions, material from pellets P1, P2 and P3 was fixed in 0.01M Na-phosphate buffer, 2% glutaraldehyde, 1% formaldehyde, pH 7.4. Preparation of the specimens was performed as described previously (Seeger et al., 2017). UV-VIS spectra of supernatants S0, S1, S2, and S3 were acquired on a NanoDrop ND-100 at a wavelength of 350–600 nm.

### Liquid Chromatography—Tandem Mass Spectrometry

The total protein content was determined using the Bradford Protein Assay Kit (BioRad Laboratories), with bovine serum albumin as standard. Aliquots of 20 μg were taken out for digestion and the volume was adjusted to 20 μL with a solution of 9 M urea in 20 mM HEPES buffer (Sigma Aldrich). Three replicates from wild type and knock out, respectively, were prepared. Proteins were reduced with dithiothreitol (DTT, Sigma Aldrich, working concentration 50 mM) for 15 min at 50°C and alkylated with iodoacetamide (IAA, Sigma Aldrich, working concentration 25 mM) for 15 min at room temperature in the dark. Samples were diluted with 50 mM ammonium bicarbonate (Sigma Aldrich) to a urea concentration of ∼ 2 M whereafter 1 μg trypsin (Promega, mass spectrometry grade) was added (enzyme:protein ratio 1:20). Digestion was performed at 37°C over night. Digestion was stopped by adding 35 μL (1/4 of total volume) of 2% trifluoroacetic acid (TFA), 20% acetonitrile (ACN) and 78% water. Samples were desalted using C18 spin columns (Pierce). After elution peptides were vacuum centrifuged to dryness using a Speedvac system ISS110 (Thermo Fisher Scientific).

The nanoLC-MS/MS experiments were performed using a Q Exactive Plus Orbitrap mass spectrometer (Thermo Fisher Scientific) equipped with a nano electrospray ion source. The peptides were separated by reversed phase liquid chromatography using an EASY-nLC 1000 system (Thermo Fisher Scientific). A set-up of pre-column and analytical column was used. The pre-column was a 2 cm EASYcolumn (ID 100 mm, 5 mm C18) (Thermo Fisher Scientific) while the analytical column was a 10 cm EASY-column (ID 75 mm, 3 mm, C18; Thermo Fisher Scientific). Peptides were eluted with a 150 min linear gradient from 4 to 100% acetonitrile at 250 nL min^–1^. The mass spectrometer was operated in positive ion mode acquiring a survey mass spectrum with resolving power 70,000 (full width half maximum), m/z 400–1,750 using an automatic gain control (AGC) target of 3 × 10^6^. The 10 most intense ions were selected for higher-energy collisional dissociation (HCD) fragmentation (25% normalized collision energy) and MS/MS spectra were generated with an AGC target of 5 × 10^5^ at a resolution of 17,500. The mass spectrometer worked in data-dependent mode.

MS raw files were processed using MaxQuant software (Cox and Mann, 2008) and the Andromeda search engine (Cox et al., 2011) against the genomes of *T. immobilis* strain MBLW1 and *E. coli* K-12 MG1655. Both genome FASTA files are deposited under their respective PRIDE accession IDs (*E. coli*: PXD022526, *T. immobilis*: PXD022559). False discovery rate (FDR) was calculated based on reverse sequences from the target-decoy search and an FDR of 1% was accepted for protein and peptide identification, with a minimum peptide length of seven amino acids. The first search was performed setting the precursor mass tolerance to 20 ppm, whereas in the second search it was lowered to 4.5 ppm, choosing a mass tolerance of 0.5 Da for the fragments. Trypsin was selected as the digestion enzyme, allowing 2 maximum missed cleavage sites. Carbamidomethylation of cysteine residues was set as static modification, while oxidation of methionine and acetylation of N-terminal were set as variable modification. In different cases, carbamylation of arginine and lysine was included as variable modification, and acetylation of N-terminal was excluded. For protein quantification, LFQ intensities were used and only proteins with at least two identified peptides were included (min. 1 unique peptide and min. 1 razor + unique peptide). Data analysis and visualization were based on LFQ intensities for proteins that were identified in three biological replicates in at least one subcellular fraction.

### Identification of Cell Surface Motif

The planctomycetes-specific cell surface signal peptide reported for *R. baltica* (Studholme et al., 2004) (Pfam: PF07595) was used to identify an equivalent motif in *T. immobilis* by searching for the consensus sequence in *T. immobilis* proteins while allowing up to 4 mismatches. Matching sequences were aligned against the seed sequences used to generate the Pfam Hidden Markov Model (HMM), and the alignment was used to create an HMM using HMMER3 (Eddy, 2009).

### Data Analysis and Visualization

The genomes of *T. immobilis* MBLW1 (GenBank: LR593887.1) and *E. coli* K12 MG1655 (GenBank: U00096.3) were re-annotated using Prokka v1.14.5 (Seemann, 2014) to allow for easier comparability of the results. The gene names and gene products from Prokka were assigned to the matching coding sequences from the original annotations. For comparison with the membrane proteome of *G. obsuriglobus* (Sagulenko et al., 2017), the obsolete NCBI reference sequence IDs were associated to the protein and gene IDs of the *G. obscuriglobus* GenBank assembly LR593888.1.

Transmembrane proteins were predicted using Phobius 1.01 (Käll et al., 2004, 2007) and the TMHMM v2.0 Server (Sonnhammer et al., 1998; Krogh et al., 2001). The presence of type I signal peptides was predicted using the SignalP-5.0 Server (Almagro Armenteros et al., 2019). Subcellular protein localization was predicted by using the PSORTb v3.0.2 Server (Yu et al., 2010). Subcellular localization predicted by PSORTb was only reported for proteins that had an unambiguous prediction, meaning that proteins with predicted multiple subcellular localizations were summarized in the category “Unknown.” Lipoproteins were predicted using the LipoP 1.0 Server (Juncker et al., 2003). Beta-barrel outer membrane proteins were predicted using BOMP (Berven et al., 2004). Functional classification was based on the Clusters of Orthologous Groups, COGs (Tatusov et al., 2000).

For visualization in heatmaps, LFQ values were first normalized by the maximum value in each replicate. A normalized mean was then calculated for each protein, by first calculating the median of all non-zero replicates in each fraction, and then taking the average of the non-zero fraction medians. For each replicate, the deviation in percent from this normalized mean was plotted.

### Phylogenetic Analysis

For the phylogeny and domain architecture analysis of the proteins in *Planctomycetes* and other bacteria, signal peptides and transmembrane domains were annotated using SignalP-5.0 and Phobius 1.01. Conserved domains were assigned to the proteins using the pfam_scan.pl script with a minimum sequence-evalue of 0.01 and the Pfam 32.0 database (Punta et al., 2012). The proteins were aligned using the mafft-linsi alignment algorithm in MAFFT v7.310 (Katoh et al., 2005), and a maximum likelihood phylogeny was inferred using LG + Γ amino acid substitution model with 100 bootstraps in RAxML version 8.0.26 (Stamatakis, 2014).

Protein BLAST (Altschul et al., 1997, 2005) was used to find homologs to GMBLW1_25620 in the refseq_select database (Update date: 2020/12/13) using default settings. The protein sequences were aligned with MAFFT L-INS-i (v7.245) (Katoh and Standley, 2013) and trimmed with TrimAL (v1.4.rev15, with the option –gt 0.5) (Capella-Gutiérrez et al., 2009). A phylogenetic tree was built using IQ-TREE (version 1.6.12, with the options -m MFP -bnni –bb 1000 –alrt 1000) (Minh et al., 2020). Using the option model finder in IQ-TREE the selected model was LG + F + I + G4 chosen according to the Bayesian information criterion. Finally, the tree was edited in TreeGraph2 (Stöver and Müller, 2010) and UFBoot (Hoang et al., 2018) used to display the branch support in the figure.

## Results

### FIB-SEM-Based 3D-Reconstruction Demonstrates Continuous but Heavily Invaginated Membrane Network in *T. immobilis*

**FIGURE 1:**
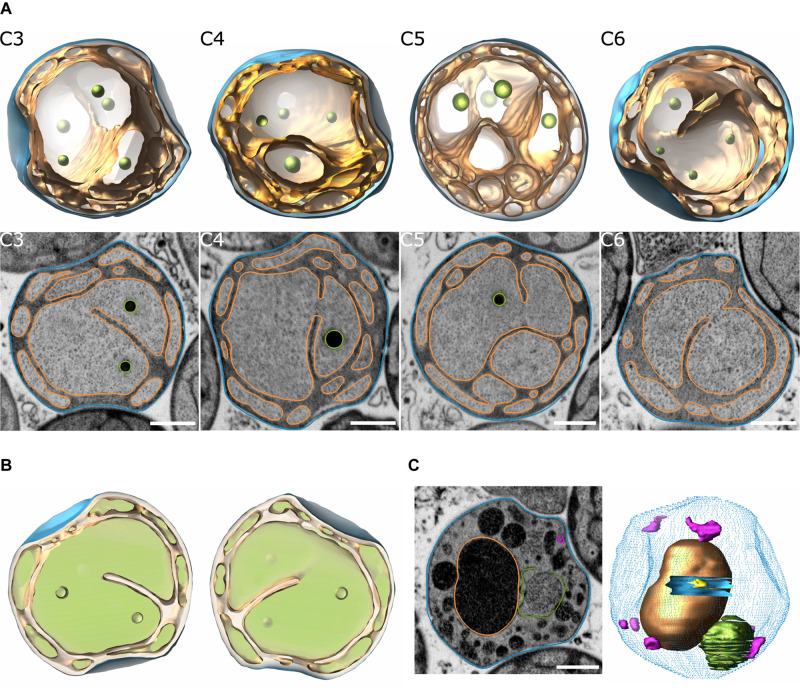
Reconstruction of *T. immobilis* cell structures based on FIB-SEM analysis. **(A)** Snapshots of 3D-model (top) and segmented SEM micrographs (bottom) of 4 manually segmented cells (“C3,” “C4,” “C5,” “C6”) depicting the cytoplasmic membrane (orange) and cell envelope (blue). Scale bar: 500 nm. **(B)** “Filled” model view of cell “C3” at y = 0° and y = 180° to illustrate cytoplasmic space (light green) and periplasmic space (light orange). **(C)** Snapshot of segmented SEM micrograph (left) and partially reconstructed model (right) of cell displaying signs of membrane damage and lysis (blue dotted contour: cell envelope; blue solid mesh: membrane hole; pink mesh: empty membrane vesicles; green: ruptured membrane vesicle). Scale bar: 500 nm.

Three-dimensional (3D) models of *T. immobilis* were reconstructed based on FIB-SEM analysis. Three cells were fully reconstructed and one additional cell was partially reconstructed (Figure 1A, Supplementary Figures 1–4, Supplementary Movies 1–4, and Supplementary Table 1). The reconstructed cells showed the presence of a tunnel-system made by invaginations of the cytoplasmic membrane, which divides the non-compartmentalized cytoplasmic space from the enlarged periplasmic space (Figure 1B). This cell architecture was also observed in several other visually inspected cells of the prepared volume. *T. immobilis* cells with apparent enclosed structures in the cytoplasmic space displayed clear signs of membrane damage, intracellular debris, ruptured membrane vesicles and a low-contrast periplasmic space (Figure 1C, Supplementary Figures 5, 6, and Supplementary Movie 5). Such seemingly damaged cells were not considered representative of the cell plan of healthy and intact cells.

As a control, 3D-models of *G. obscuriglobus* were reconstructed using the same methodology. The overall cell architecture was similar to the one observed in *T. immobilis*. However, the nucleoid in *G. obscuriglobus* is more condensed and visible by FIB-SEM and other electron microscopy techniques and could therefore be reconstructed. In the reconstructed single cell, the nucleoid appeared to consist of one DNA-complex, while the invaginated membranes and the condensed nucleoid were present in both mother and daughter cells of a budding cell pair that was captured at a late budding stage (Supplementary Figure 7). This is consistent with earlier findings showing that neither invaginated membranes nor DNA were observed early in the budding process (Santarella-Mellwig et al., 2013), but only at a later stage (Lee et al., 2009). As in a previous study using cryo electron tomography (Mahajan et al., 2020a), we observed a single, spherical particle of up to 400 nm in diameter in almost every cell. In the reconstructed and another, visually inspected, budding cell, the particle was only present in the mother cell.

### Differential Solubilization of *T. immobilis* Results in Biochemically, Physicochemically and Morphologically Distinct Cell Fractions

**FIGURE 2:**
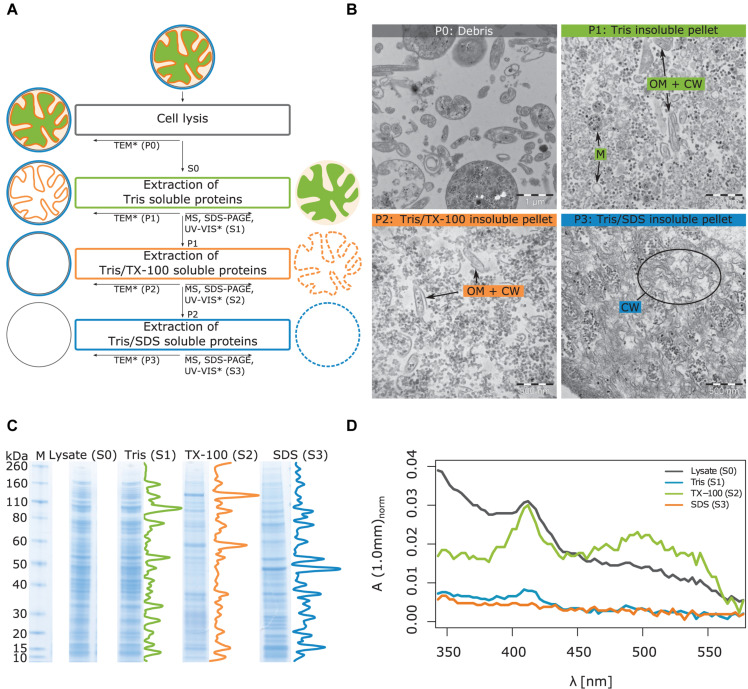
Illustration of workflow for subcellular fractionation by differential solubilization and results from imaging and biochemical analysis of the subcellular fractions. **(A)** After lysis, unbroken cells and debris are removed by low-speed centrifugation (P0). The Tris-soluble proteins (S1) are separated from the Tris-insoluble pellet (P1) by ultracentrifugation. The Tris/TX-100-soluble proteins (S2) are further separated from the Tris/TX-100-insoluble pellet (P2) by ultracentrifugation. In the last step, Tris/SDS-soluble proteins (S3) are separated from the Tris/SDS-insoluble pellet (P3). Supernatants and pellets were subjected to the indicated analyses: TEM, Transmission Electron Microscopy, MS, LC-MS/MS, Liquid chromatography coupled to tandem-mass-spectrometry, SDS-PAGE, UV-VIS spectroscopy. *Method applied only to *T. immobilis*. **(B)** Analysis of *T. immobilis* pellets P0, P1, P2, and P3 by TEM. OM: outer membrane; CW, cell wall; M, cytoplasmic membrane. **(C)** Analysis of subcellular fractions (S1, S2, S3) and the total cell lysate (S0) by SDS-PAGE (M: marker with corresponding molecular weights in kDa). Density profiles are shown next to the lanes of the subcellular fractions. The corresponding SDS-PAGE analysis for *E. coli* is shown in Supplementary Figure 9. **(D)** Analysis of subcellular fractions (S1, S2, S3) and total cell lysate (S0) from *T. immobilis* by UV-VIS spectroscopy. The absorbance was normalized with respect to the total protein concentration of each fraction.

To determine the protein content of the cytoplasmic and membrane fractions of the cell, *T. immobilis* was cultivated in the laboratory until the bacterial cells reached early stationary phase. The generation time was estimated to g = 8 h (Supplementary Figure 8A). *E. coli* K12 MG1655 was included as a control and likewise cultivated until early stationary phase (Supplementary Figure 8B; g = 35 min). Following cell lysis by sonication, a series of protein extractions were performed, as schematically shown in Figure 2A. Subcellular fraction S1 contained proteins that are soluble in Tris, fraction S2 contained proteins that are soluble in Tris/Triton X-100 (TX-100) and fraction S3 proteins that are soluble in Tris/SDS. For *T. immobilis*, as much as 64% of the cell lysate were recovered in the S1 fraction, as compared to 8% in the S2 fraction and 1% in the S3 fraction (Supplementary Table 2) adding up to an overall yield of 75%.

Imaging analysis of the different *T. immobilis* subcellular fractions was performed by transmission electron microscopy (TEM) to get a visual overview of the fractionation procedure (Figure 2B). The pellet P0 (Debris) contained predominantly partially lysed cells that display spiral “Brezel”-like structures, and we also noted a few cells that had not been lysed. In pellet P1 (Tris insoluble fraction), there were, besides ribosomes and polysomes, two distinct structural components, small vesicles and non-spherical irregularly shaped structures. Early TEM analysis of crude envelope preparations from *E. coli* indicated that the vesicular structures represent the cytoplasmic membrane, whereas the non-spherical structures correspond to the cell envelope (outer membrane + cell wall) (Schnaitman, 1970, 1971). Thus, we hypothesize that proteins that could not be solubilized in Tris are associated with either the cytoplasmic or outer membrane or the cell wall. Tris soluble proteins can also be trapped in membrane vesicles or associated with membrane-spanning complexes and thereby also be part of pellet P1.

In pellet P2, the vesicular structures are no longer visible, suggesting that the inner membrane proteins have been solubilized in Tris/TX-100 and thus are part of the S2 fraction. However, the irregularly shaped structures of the cell envelope are still observed, in addition to ribosomes and polysomes. Pellet P3 contains neither the membrane vesicles (solubilized in S2) nor the non-spherical open structures, which suggests that the outer membrane proteins have been solubilized in the S3 fraction. The remaining structures in the TEM images are fibrous and likely to represent SDS-insoluble cell wall fragments. No unbroken or partially lysed cells were observed in the TEM images from either of pellets P1, P2 and P3. The ribosomes were mostly solubilized in the first S1 fraction, although observed in all fractions. Thus, based on the TEM analysis we conclude that the cellular components have been fractionated based on their different solubility in Tris, TX-100 and SDS.

Analysis by SDS-PAGE confirmed distinct protein profiles in the three subcellular fractions, both for *T. immobilis* (Figure 2C) and *E. coli* (Supplementary Figure 9). For *T. immobilis*, the protein profiles of the S0 (lysate) and S1 fractions were practically identical. This result agrees with the calculated protein yields, showing that approximately 65% of all proteins are soluble in Tris (Supplementary Table 2). Fractions S2 and S3 showed distinct protein profiles, which was evident from both visual inspection and from linescans of the respective lanes of the SDS-PAGE gel.

The *T. immobilis* subcellular fractions were further analyzed by UV-VIS spectroscopy (Figure 2D) utilizing the characteristic absorption pattern of heme-containing cytochromes at 410 nm and the absorption of carotenoids at a wide peak around 500 nm. The analysis of the lysate confirmed high absorption at both 410 nm and from 450 to 550 nm. The spectrum of fraction S1 displayed a minor peak at 410 nm but practically no absorption at higher wavelengths. The spectrum of fraction S2 displayed a strong peak at 410 nm and a wide peak at 500 nm, suggesting the presence of heme and carotenoids, respectively. Supernatant S3 displayed practically no absorption at 410 nm or higher wavelengths. Thus, most of the heme-containing cytochromes and carotenoids, which are located in the cytoplasmic membrane, are enriched in the S2 fraction, consistent with the hypothesis that most of the membrane proteins are present in this fraction.

### Proteomics of the Subcellular Fractions

**FIGURE 3:**
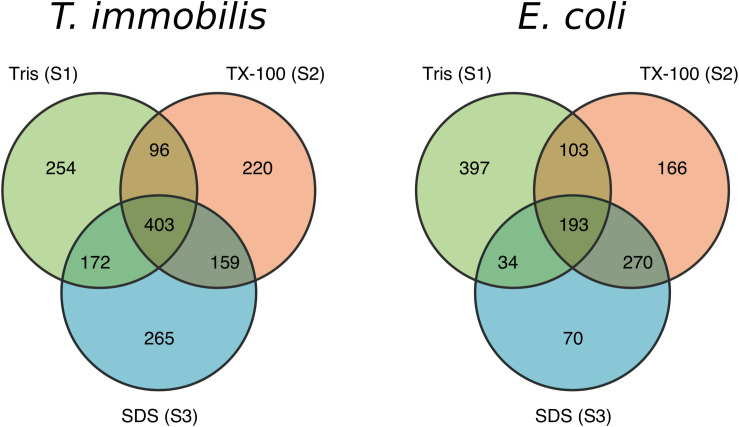
Venn diagram containing the number of unique and overlapping proteins of the three subcellular fractions in *T. immobilis* and *E. coli*; Tris soluble [“Tris (S1)”], Tris/TX-100 soluble [“TX-100 (S2)”] and Tris/SDS soluble [“SDS (S3)”].

We analyzed the protein contents of the three cell fractions using LC-MS/MS in *T. immobilis*, and also in *E. coli* as the control. Proteins that are identified at least in one sample are summarized in Supplementary Table 3. More stringent criteria were applied for a protein to be assigned to a particular fraction, which required protein identification in each of the three biological replicates of a particular fraction. Using the strict criteria, a total of 1,569 proteins were identified in *T. immobilis* and 1,233 proteins in *E. coli* (Supplementary Table 4). Of these, 739 proteins were uniquely found in a single fraction in *T. immobilis* (Figure 3), as compared to 633 such proteins in *E. coli* (Figure 3). The fraction-specific proteins were almost equally distributed among the three fractions in *T. immobilis* (220–265 proteins per fraction), whereas the number of unique proteins in each fraction differed fivefold in *E. coli* (70 proteins in fraction S3 to 397 proteins in fraction S1). Another 172 proteins were identified in both fractions S1 and S3 in *T. immobilis*, whereas only 34 proteins showed an overlap between these two fractions in *E. coli*.

### Subcellular Fractionation and Predicted Localization Patterns

The predicted subcellular locations of the proteins identified in the LC-MS/MS analyses were very similar in the two species (Supplementary Table 5). Of the proteins for which a subcellular location was assigned, 632–757 proteins were predicted to be cytoplasmic proteins. Additionally, 315–335 proteins were suggested to be inner membrane proteins and another 14–46 proteins were predicted to be outer membrane proteins.

**FIGURE 4:**
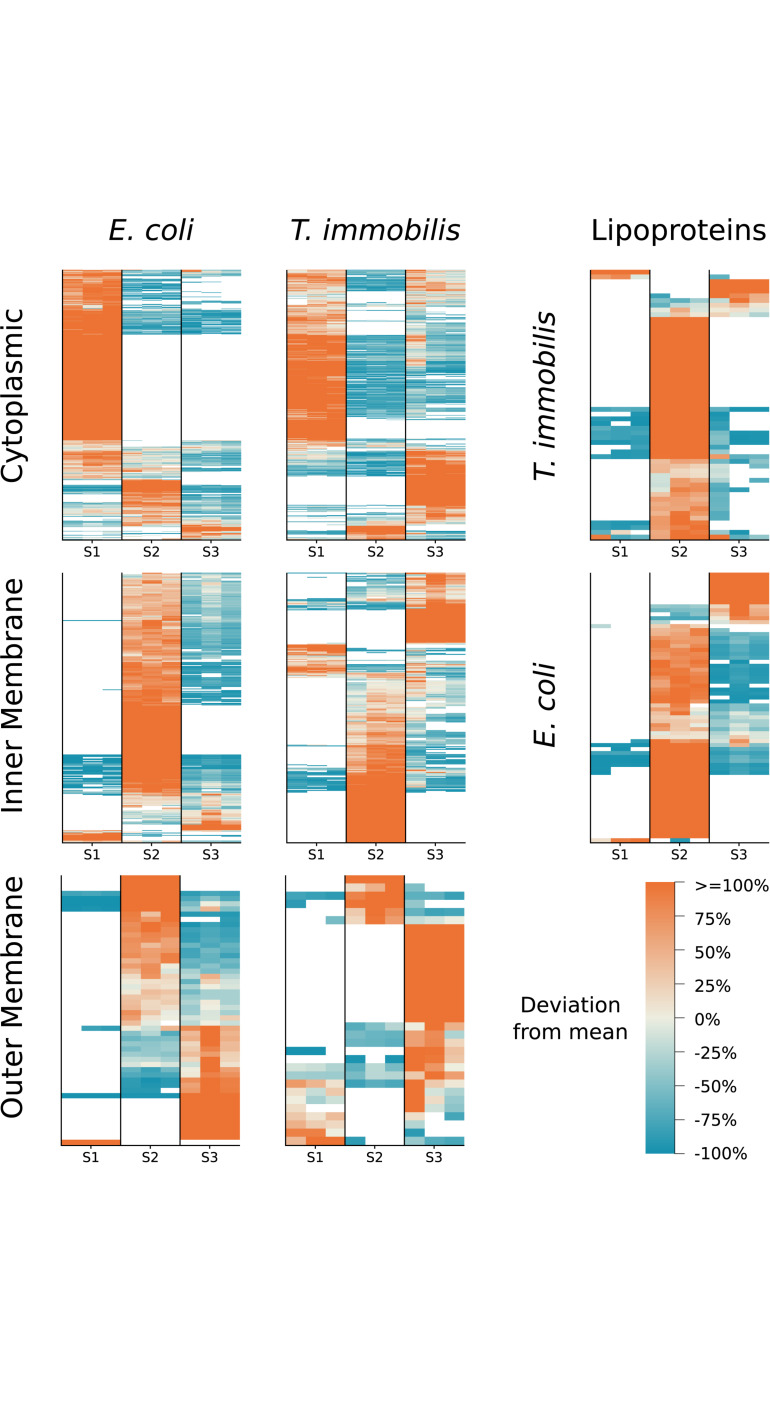
Subcellular localization predictions and relative abundance for *T. immobilis* and *E. coli* subcellular proteomes. Heatmaps for each species illustrate the relative abundance of predicted cytoplasmic, inner/cytoplasmic membrane, outer membrane proteins and lipoproteins as a function of the subcellular fraction [Tris soluble (S1), Tris/TX-100 soluble (S2), Tris/SDS soluble (S3)]. Proteins exclusively found in one fraction were colored as though they deviated from the mean by 100%. PSORTb v3.0 was used to predict cytoplasmic, inner membrane and outer membrane proteins. In addition, BOMP was used to complement outer membrane predictions. LipoP v1.0 was used to predict lipoproteins.

We compared the protein fractionation patterns with the predicted protein localization patterns (Figure 4 and Supplementary Table 6). In both species, we observed that many of the predicted cytoplasmic proteins were associated with fraction S1, while inner membrane proteins (e.g., proteins of the SEC translocon) and outer membrane proteins (e.g., BamA) were commonly found in fractions S2 and S3, respectively. Lipoproteins are characterized by a type II signal peptide which enables even hydrophilic lipoproteins to anchor to the cytoplasmic and/or the outer membrane (Babu et al., 2006; Nakayama et al., 2012). We identified 57 and 68 lipoproteins in *T. immobilis* and *E. coli*, respectively, and these were either uniquely present in or strongly enriched in fraction S2 in both species.

### Cell Fractionation Patterns of Cell Envelope Proteins

**FIGURE 5:**
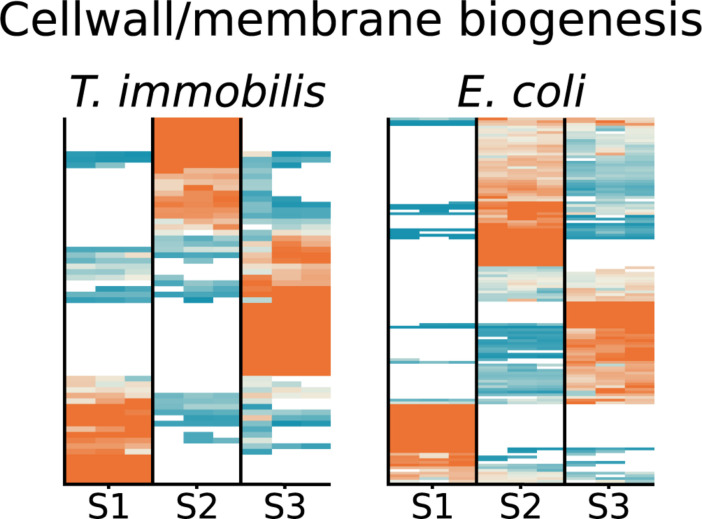
Heatmap showing the relative abundance of 65 expressed proteins in *T. immobilis* and 133 proteins in *E. coli* belonging to the Cluster of Orthologous Group (COG) category “Cell wall/envelope biogenesis” (M) as a function of the respective subcellular fraction [Tris soluble (S1), Tris/TX-100 soluble (S2), and Tris/SDS soluble (S3)]. The heatmaps are colored according to the scale shown in Figure 4.

To learn more about the fractionation patterns of cell envelope proteins, we compared the proteins sorted into the cell wall/membrane/envelope category of Clusters of Orthologous Groups (COGs) in the two species. Overall, the fractionation patterns of these proteins were similar, with cell envelope proteins identified in all subcellular fractions (Figure 5 and Supplementary Table 7). In *T. immobilis*, 65 expressed proteins were classified into this category, as compared to 133 proteins in *E. coli*. However, the large majority of these proteins were species-specific and only 17 homologous proteins were present in both species, including proteins involved in the β-barrel-assembly machinery for outer membrane proteins (BamA, BamB), membrane protein insertion (YidC), lipoprotein maturation (Lnt), and lipoprotein release (LolCDE). The BamA protein was enriched or uniquely present in fractions S2 and S3 in both species, and the sole BamB protein in *E. coli* was detected in fractions S2 and S3. The *T. immobilis* genome contains as many as 28 paralogous genes for BamB proteins (molecular weights range from 43 to 193 kDa, pI values from 5.1 to 9.3), of which 3 were exclusively identified in fraction S1 (bamB_4, bamB_7, bamB_28), one in fraction S2 (bamB_17) and one in fraction S3 (bamB_1).

Also included in the set of homologs were genes for the biosynthesis of lipopolysaccharides (KdsA, LpxK, RfbB, and WaaA). The cytoplasmic enzyme KdsA, which is involved in the synthesis of core oligosaccharide Kdo, was identified in fraction S1 in both species, whereas the Kdo transferase, WaaA, which ligates the sugar subunits of the polysaccharide to the Kdo-lipidA was enriched in fraction S3. LpxK, which is required for the phosphorylation of lipidA was exclusively identified in fraction S3 in *T. immobilis*, while it was present in all fractions in *E. coli*. In accordance with previous studies from other *Planctomycetes* species, these results suggest that *T. immobilis* has a bacterial-like outer membrane assembly machinery and lipopolysaccharide.

### Pilins and the SBP_bac_10 Domain Proteins

The prepilin cleavage motif, also described as the “N_methyl” Pfam domain, is recognized by the peptidase GspO/PilD and functions as a signal to transport the pilin proteins to the cytoplasmic membrane during the assembly of Type 2 Secretion Systems (T2SS) and Type 4 Pili (T4P) protein complexes. The prepilin cleavage motif was found in a family of heavily expanded proteins encoded by 41 or more genes in each genome of the *Planctomycetales* (Mahajan et al., 2020b), most of which also contained the “SBP_bac_10” Pfam domain. We identified 31 of the 100 proteins in *T. immobilis* that carry the “N_methyl” Pfam domain, and 25 of the 91 proteins with both domains (Supplementary Table 8). The genes encoding proteins with both domains were evenly spread around the genome of *T. immobilis*, and often flanked by genes containing type-I or type-II signal peptides.

**FIGURE 6:**
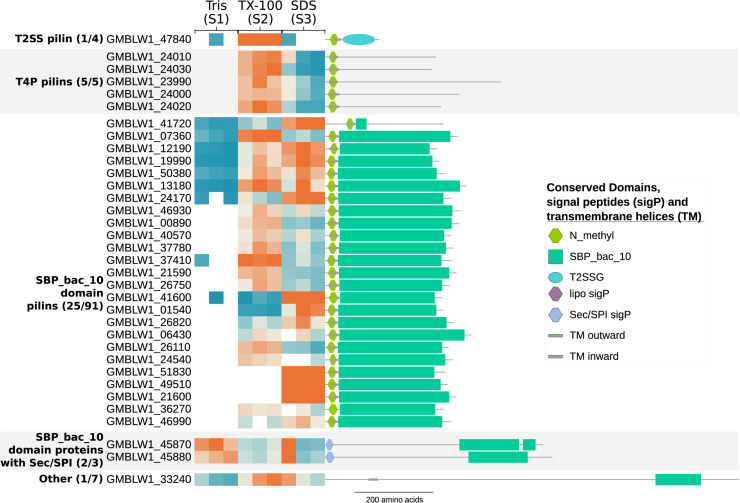
Subcellular fractionation profiles and domain architectures of protein groups in *T. immobilis* with N_methyl prepilin cleavage motif and/or SBP_bac_10 Pfam domains. The numbers in parenthesis for each protein group represent the number of identified proteins out of the total number of predicted proteins in the group. The heatmap shows the relative abundance of the experimentally identified proteins (colored according to Figure 4) as a function of the respective subcellular fraction [Tris soluble (S1), Tris/TX-100 soluble (S2), and Tris/SDS soluble (S3)]. Signal peptides were predicted with SignalP-5.0 and transmembrane domains were predicted using Phobius 1.01.

The results revealed a striking correlation between the domain architectures and the fractionation patterns of these proteins (Figure 6). Thus, pilin proteins with the “N_methyl” Pfam were strongly enriched in fraction S2, whereas proteins with both domains were mostly found in both fractions S2 and S3. Two of the three proteins with the Sec/SPI signal peptide and the “SBP_bac_10” Pfam domain plus the single identified protein with only an “SBP_bac_10” Pfam domain were present in all cell fractions. Finally, proteins with Sec/SPI or lipoprotein signal peptides combined with other SBP_bac_^∗^ domains (where ^∗^ is a number) were identified in fraction S2 in *T. immobilis*, whereas such proteins in *E. coli* were recovered from fraction S1, S2 or both S1 and S2 (Supplementary Figure 10 and Supplementary Table 8).

### Cell Surface Proteins

**FIGURE 7:**
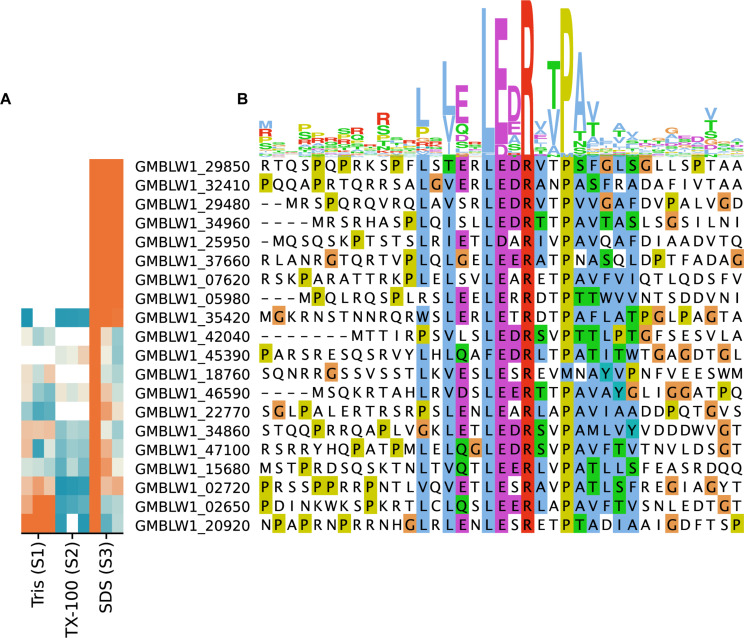
Sequence logo and subcellular fractionation patterns of proteins in *T. immobilis* with *Planctomycetes-*specific signal peptides. **(A)** Heatmap showing the relative abundance of 20 expressed proteins (colored according to Figure 4), each carrying the *Planctomycetes* specific signal peptide as a function of the respective subcellular fraction [Tris soluble (S1), Tris/TX-100 soluble (S2), and Tris/SDS soluble (S3)]. **(B)** Sequence alignment of experimentally identified proteins in *T. immobilis* carrying a *Planctomycetes*-specific signal peptide. The sequence logo is shown on top of the aligned sequences.

The consensus motif RRLxxExLExRxLLA identified in cell surface proteins in *R. baltica* is thought to represent a novel N-terminal export signal peptide (Studholme et al., 2004). We identified 52 genes coding for proteins with the LxLExLExRxxP motif in the *T. immobilis* genome, of which 20 were detected in the proteomics data sets and 8 of these were exclusively present in fraction S3 (Figure 7 and Supplementary Table 9). Identifiable Pfam domains were found in 43 of the 52 proteins, including 17 of the 20 expressed proteins (Supplementary Table 9). In this set, we identified two proteins with N-terminal trypsin domains in serine proteases, one protein with an N-terminal glucose dehydrogenase domain and one protein with 7 bacterial immunoglobulin-like domains. Additionally, we identified two proteins with integrin-beta domains in Na/Ca exchangers. This domain was also identified in proteins not identified as expressed in our analysis, including a > 30 kb and a > 40 kb long gene encoding more than 30 and 12 integrin beta domains, respectively. Interestingly, 8 of the 10 longest proteins encoded by the *T. immobilis* genome, ranging in size from 4,016 to 10,566 amino acids and from 287 kDa to 1.1 MDa, have the novel signal peptide motif and are likely to be surface exposed proteins.

### Peptidoglycan Biosynthesis and Cell Division Proteins

In *T. immobilis*, most genes for the biosynthesis of a conventional peptidoglycan could not be identified (Mahajan et al., 2020a; Wiegand et al., 2020), but dehydrogenases (WbpA and WcaJ) and transferases (WbpU) that catalyze the conversion of UPD-GlcNac to UDP-GlcNacA or to UDP-GalNac, of which WbpA were exclusively found in fraction S3. These nucleotide-sugar compounds serve as glycosyl donors to polysaccharides, proteins or lipids in reactions that are catalyzed by glycosyl transferases.

In *E. coli*, proteins involved in the biosynthesis of the peptidoglycan cell wall accounted for many of the unique proteins in this species, with the cytoplasmic proteins involved in this pathway enriched in fraction S1, while Mur J, the lipid II flippase, was recovered in fraction S3 (Supplementary Table 3). The Braun lipoprotein, Lpp, which anchors the outer membrane to the peptidoglycan cell wall in *E. coli* was recovered from fraction S3 in *E. coli*. In addition, a wide variety of peptidoglycan glycosyltransferases, transpeptidases, and murein transglycosylases were identified. Proteins in cell elongation and division complexes that are coupled with peptidoglycan synthesis and chromosome segregation were also identified among the expressed proteins in *E. coli* (e.g., MreB, RodZ, FtsABEIKLNQWXZ, MinCDE, ZapAD, ZipA, MukBEF). These results show that proteins for peptidoglycan biosynthesis, cell elongation and cell division can be recovered in the cell fractionation study if such proteins were present.

### Comparison to Proteins Identified in Cell Wall Preparations

**FIGURE 8:**
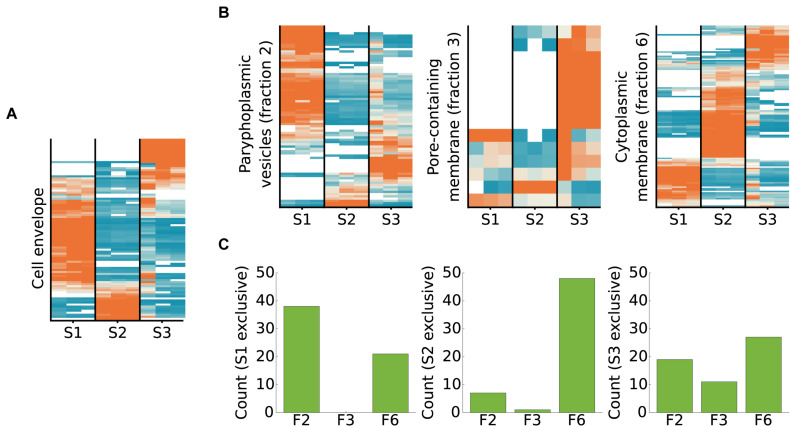
Subcellular fractionation profiles of *T. immobilis* cell envelope proteins and homologs of proteins identified in membrane proteomes of *G. obscuriglobus*. **(A)** Relative abundance and subcellular fractionation profiles of 89 proteins from the cell wall proteome of *T. immobilis* (Mahajan et al., 2020a). **(B)** For proteins unique to each *G. obscuriglobus* membrane fraction (paryphoplamsic vesicles (fraction 2), nuclear-pore containing membrane (fraction 3) and cytoplasmic membrane fraction (fraction 6) (Sagulenko et al., 2017), the relative abundance of identified *T. immobilis* homologs is shown as a function of the respective subcellular fraction [Tris soluble (S1), Tris/TX-100 soluble (S2), and Tris/SDS soluble (S3)]. Heatmaps in **(A)** and **(B)** are colored according to scale in Figure 4. (**C**) Barplot illustrating the number of fraction-specific proteins from *T. immobilis* [Tris soluble (S1), Tris/TX-100 soluble (S2), and Tris/SDS soluble (S3)] as a function of the membrane fraction of the corresponding homologs in *G. obscuriglobus* (F2: paryphoplamsic vesicles, F3: nuclear-pore containing membrane, F6: cytoplasmic membrane fraction).

We compared the proteins identified in this study to the proteins identified in a previous mass spectrometry analysis of a cell wall preparation from *T. immobilis* (Mahajan et al., 2020a). In total, 131 proteins were identified in the cell wall preparation study, of which 89 were also detected in this study (Figure 8A and Supplementary Table 10). The two cysteine-rich proteins with YTV motifs that scored the highest in the cell wall preparation were exclusively identified in fraction S3. The third highest scoring protein in the cell wall proteome, GMBLW1_25620, which is predicted to be a lipoprotein with a type II signal peptide (as identified by both LipoP and SignalP-5.0), was also uniquely recovered from fraction S3. Phylogenetic analysis demonstrates that this protein is solely present in the *Planctomycetales* (Supplementary Figure 10), and it thus has a phyletic distribution profile identical to the two cysteine-rich cell wall proteins (Mahajan et al., 2020a). Most homologous proteins to GMBLW1_25620 have a molecular weight between 93 and 158 kDa and are predicted to contain either type-I or type-II signal peptides (Supplementary Table 11). Notable exceptions are two *G. obscuriglobus* homologs (WP_109571231.1, WP_010034540.1), which have a molecular weight of only 15 kDa. Also, the top-8 highest scoring proteins were among the small set of proteins exclusively identified or enriched in fraction S3 in the cell wall fraction, and the top-13 highest scoring proteins contain a type I or type II/Sec signal peptide.

### Comparison to *G. obscuriglobus* Membrane Layer Proteins

We also compared the fractionation patterns of homologs in *T. immobilis* to proteins from *G. obscuriglobus*, which have been identified in a previous mass spectrometry analysis (Sagulenko et al., 2017). Those proteins were identified from different membrane layers, referred to as paryphoplasmic (“F2”) nuclear-pore (“F3”) and cytoplasmic membrane (“F6”). More than 60% of the proteins unique for fractions F2 and F6 in *G. obsuriglobus* have homologs in *T. immobilis*, as compared to only 36% of the proteins specific to fraction F3 (Supplementary Table 12). Homologs to the *G. obscuriglobus* proteins identified in the suggested paryphoplasmic fraction F2 were mostly found in our Tris-soluble fraction S1 in *T. immobilis*, and vice versa (Figures 8B,C and Supplementary Table 13). Likewise, the majority of proteins in the suggested cytoplasmic membrane fraction F6 in *G. obscuriglobus* were mostly recovered from our fraction S2, and vice versa.

Out of the 39 proteins solely found in the pore-containing membrane fraction F3 in *G. obscuriglobus*, only 14 had expressed homologs in our dataset, including 4 predicted beta barrel proteins, 3 proteins with *Planctomycetes* specific cell surface motifs, and two lipoproteins (according to SignalP-5.0). Seven of these proteins were uniquely identified in fraction S3 in *T. immobilis*. The converse comparison showed a different pattern; homologs to the proteins uniquely found in our fraction S3 were mostly found in the presumed paryphoplasmic membrane fraction 2 and the inner membrane fraction 6 in *G. obscuriglobus*. Thus, our fraction S3 proteins did for the most part not correspond to the proteins isolated from the pore-containing membrane in *G. obscuriglobus*. Rather, the majority of proteins uniquely identified in the pore-containing membrane layer were solely identified in *G. obscuriglobus*.

### Functional Categorization of Cytoplasmic Proteins in Cell Fraction S3

**FIGURE 9:**
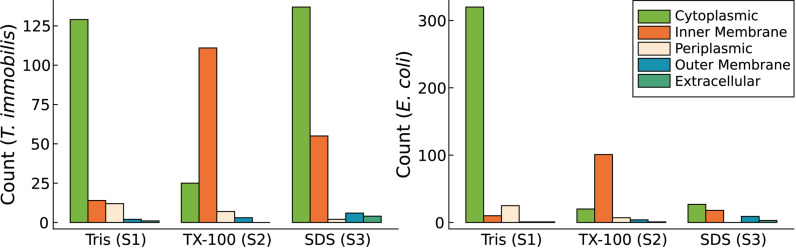
Subcellular localization predictions for proteins exclusively identified in either Tris soluble [“Tris (S1)”], Tris/TX-100 soluble [“TX-100 (S2)”], or Tris/SDS soluble [“SDS (S3)”] fractions in *T. immobilis* and *E. coli*; C: cytoplasmic; IM: inner membrane; P, periplasmic; OM, outer membrane; E, extracellular. Subcellular localization predictions were performed using PSORTb v3.0. In addition to the PSORTb v3.0 predictions, outer membrane proteins were predicted using BOMP.

We detected a surprisingly large number of cytoplasmic proteins exclusively in fraction S3 in *T. immobilis*. A total of 137 predicted cytoplasmic proteins, corresponding to more than 50% of the cytoplasmic proteins were uniquely identified in fraction S3 in all replicates (Figure 9). In contrast, only 27 of the more than 300 predicted cytoplasmic proteins in *E. coli* were exclusively identified in fraction S3 (Figure 9).

**FIGURE 10:**
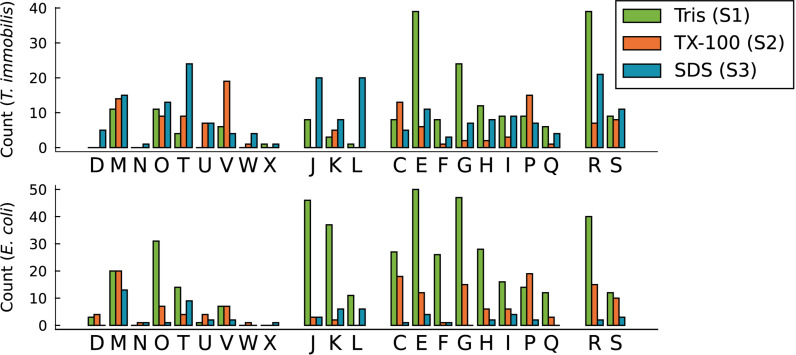
Functional analysis of *T. immobilis* and *E. coli* subcellular proteomes based on COGs. The number of fraction-specific proteins is shown as a function of the COG category for the proteomes of *T. immobilis* and *E. coli*. One-letter abbreviations and full names of COG categories: Amino acid transport and metabolism (E), carbohydrate transport and metabolism (G), cell cycle control, cell division, chromosome partitioning (D), cell motility (N), cell wall/membrane/envelope biogenesis (M), chromatin structure and dynamics (B), coenzyme transport and metabolism (H), defense mechanisms (V), energy production and conversion (C), extracellular structures (W), function unknown (S), general function prediction only (R), inorganic ion transport and metabolism (P), intracellular trafficking, secretion, and vesicular transport (U), lipid transport and metabolism (I), mobilome: prophages, transposons (X), nucleotide transport and metabolism (F), posttranslational modification, protein turnover, chaperones (O), RNA processing and modification (A), replication, recombination and repair (L), secondary metabolites biosynthesis, transport and catabolism (Q), signal transduction mechanisms (T), transcription (K), translation, ribosomal structure, and biogenesis (J).

To learn more about the biased distribution of cytoplasmic proteins we sorted all proteins exclusively identified in a single fraction in all three replicates according to their COG categories (Figure 10). The results indicated that the main difference in the fractionation patterns between the two species concerned proteins involved in signal transduction (T) and basic information processing, such as translation (J), transcription (K) and replication (L). Proteins classified into these functional categories were mostly associated with fraction S1 in *E. coli*, as expected, whereas a surprisingly large fraction of proteins in these categories were identified in fraction S3 in *T. immobilis* (Supplementary Tables 14,15). Proteins of general (R) or unknown (S) function were also relatively more often identified in fraction S3 in *T. immobilis* (Figure 10).

**FIGURE 11:**
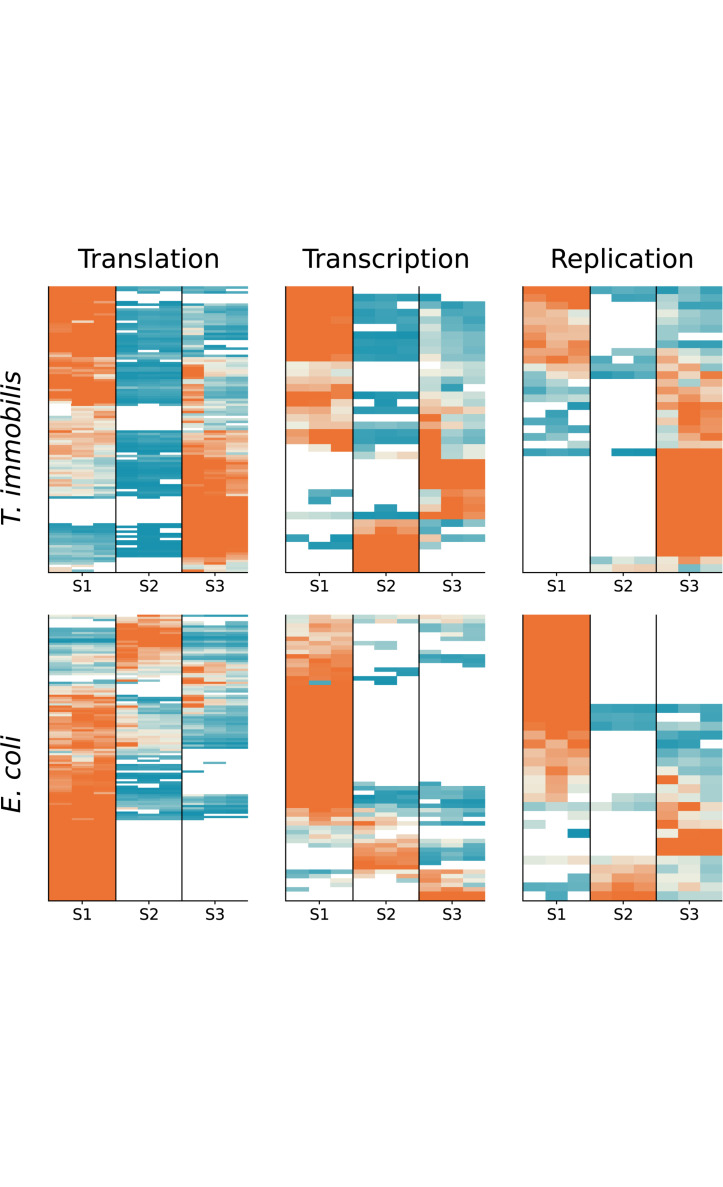
Subcellular fractionation profiles of *T. immobilis* and *E. coli* proteins involved in information storage and processing. The relative abundance of proteins (colored according to Figure 4) related to COG categories Translation (“J”), Transcription (“K”) and Replication (“L”) are shown for a given subcellular fraction [Tris soluble (S1), Tris/TX-100 soluble (S2), and Tris/SDS soluble (S3)].

We compared the fractionation patterns of all proteins sorted into the transcription, replication and translation categories in *T. immobilis* and *E. coli* (Figure 11). The results confirmed that several proteins in the replication, transcription and translation categories were uniquely associated with fraction S3 in *T. immobilis*, with a correspondingly large number of proteins in these categories uniquely associated with fraction S1 in *E. coli*.

### Novel Signal Transduction Proteins and Regulatory Signals

**FIGURE 12:**
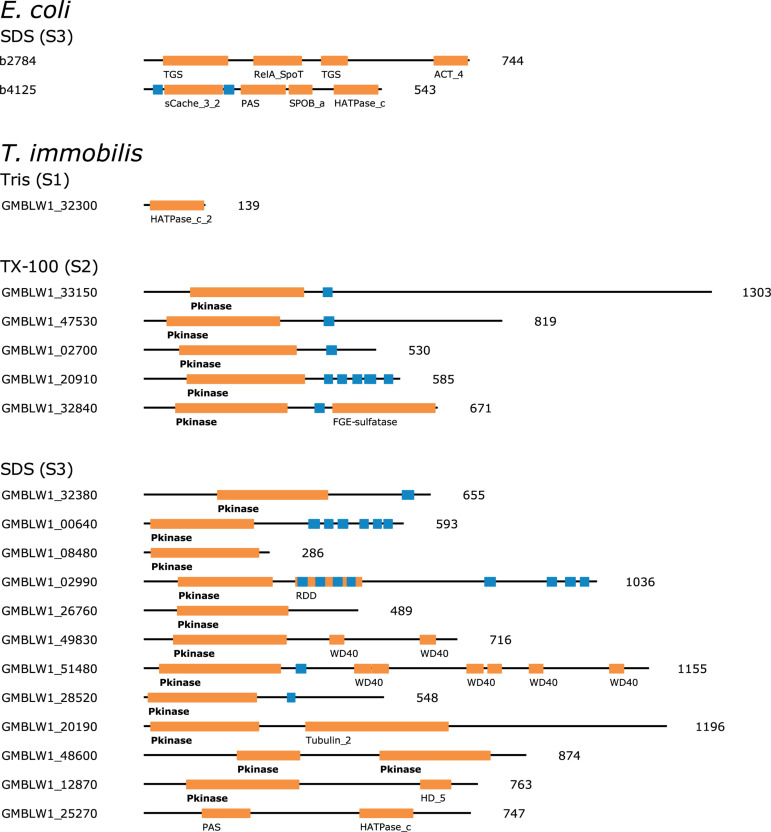
Domain architecture of fraction-specific proteins with kinase domains identified in the subcellular proteomes of *T. immobilis* and *E. coli*. The Pfam domains are highlighted with orange boxes and the respective domain ID. Blue boxes indicate the location of predicted transmembrane domains based on Phobius 1.01. The protein lengths are indicated besides each domain scheme.

The large majority of proteins involved in signal transduction processes (T) in *T. immobilis* were identified in fraction S3 (S1: 4, S2: 9, S3: 24). Histidine kinases are the major bacterial signaling systems. The histidine kinase protein DcuS, which contains the domains PF00989 and PF02518, was identified in fraction S3 in both *E. coli* and *T. immobilis*. Serine/Threonine kinases are one of the major signaling systems in eukaryotes, but less common in bacteria. However, major expansions and diversification of serine/threonine kinases have been reported in the *Planctomycetes* (Arcas et al., 2013; Mahajan et al., 2020b). Interestingly, about 50% of the proteins classified into the signal transduction category and recovered from fractions S2 and S3 in *T. immobilis* contained at least one Pkinase domain (Pfam ID: PF00069), commonly found in Serine/Threonine kinases (Figure 12). The Pkinase domain was linked to a variety of other domains in these proteins. Thus, *T. immobilis* has a unique set of Serine/Threonine kinases that are not soluble in either Tris or Triton X-100 and for which no homologs are present in *E. coli*. Interestingly, four expressed phosphoserine phosphatases are also associated with fractions S2 and S3, and two of these (RsbU_2 and RsbU_3) are uniquely found in fraction S3. Thus, much of the difference in the fractionation patterns of signal transduction proteins between the two species was related to the serine/threonine kinases in *T. immobilis*.

Another notable difference between the two species was the identification of 38 proteins in the transcription category uniquely present in fraction S1 in *E. coli*, of which 27 represented various different transcriptional regulators, as compared to the presence of only one transcriptional regulator in fraction S1 in *T. immobilis*. Instead, two paralogs of the major sigma factor, SigA_1 and SigA_2 were uniquely present in fraction S3 in *T. immoblis*. Furthermore, two of the extracytoplasmic (ECF) sigma factors SigR_1 and EcfG, were exclusively present in fraction S2 and another three, SigE, SigL, and SigW were only recovered in a single experiment in either fraction S2 or S3. Previous studies have shown that most of the ECF sigma factors are associated with multiple WD40 beta-sheet repeat domains, which in eukaryotes are involved in protein-protein interactions, such as in signal transduction pathways.

Also identified in fraction S3 in *T. immobilis* was RNA polymerase associated protein, RapA, while being identified in fraction S1 in all replicates in *E. coli.* We identified as many as five copies of these genes in *T. immobilis* and several more copies were found in several other *Planctomycetes* species. The RapA protein is an RNA polymerase recycling factor, which interacts with the post-transcription complex to outcompete the sigma factor, thereby enabling release of the RNA polymerase following transcription. In eukaryotes, the RapA proteins belong to the Swi2/Snf2 protein family and form a multi-subunit complex that makes tightly packaged DNA more accessible to RNA polymerase and transcription factors (Jin et al., 2011). The RapA protein in *E. coli* contains only the RapA_C domain, while all homologs in *T. immobilis* contain the SNF2_N domain and the Helicase_C domain (Supplementary Figure 12). The two RapA proteins expressed in the S3 fraction in *T. immobilis* have thus a different domain structure than the single *E. coli* RapA protein expressed in the S1 fraction.

These results suggest that proteins involved in signal transduction pathways, including serine/threonine kinases as well as ECF sigma factors and proteins that enable recycling of sigma factors are associated with fraction S3 in *T. immobilis*, whereas the large majority of transcriptional regulators in *E. coli* are cytoplasmic and thereby recovered from fraction S1. The difference in fractionation patterns may thus reflect the increased need for molecular systems to flexibly transmit signals from the exterior to the interior of the complex *Planctomycetes* cells.

### Enzymes for the Degradation, Modification and Repair of Nucleic Acids

An inspection of the functional annotations for fraction S3-specific proteins in the replication and translation categories showed that the different fractionation patterns were largely attributed to enzymes that degrade, modify or repair DNA and RNA molecules, whereas proteins involved in the synthesis of DNA, RNA, and proteins were enriched in fraction S1 in both species. We examined the domain structures and performed phylogenetic inferences of several cytoplasmic proteins for these processes that were identified in fraction S3, as described below.

**FIGURE 13:**
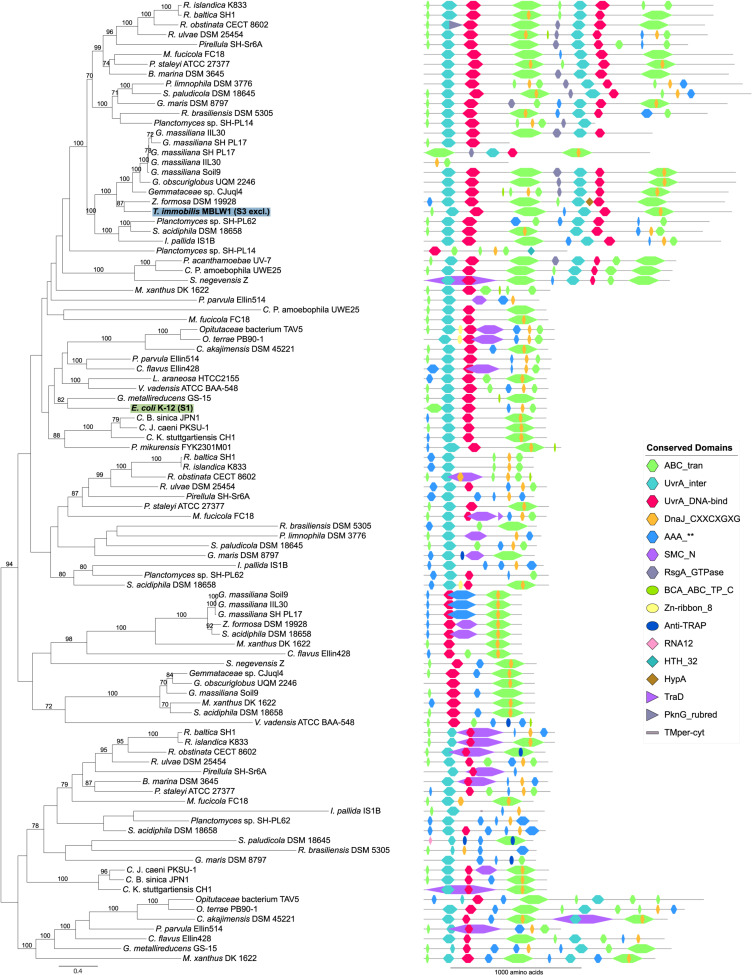
Phylogeny and domain architecture of UvrA in *Planctomycetes* and other bacteria. The species names of the proteins expressed in the subcellular fractionation assay are shown in bold. The corresponding subcellular fractions are mentioned in parenthesis and further highlighted by colored boxes [green: Tris (S1), blue: SDS (S3)]. Signal peptides and transmembrane domains were annotated using SignalP-5.0b and Phobius 1.01. Conserved domains were assigned to the proteins using the pfam_scan.pl script with a minimum sequence-evalue of 0.01 and the Pfam 32.0 database. The proteins were aligned using the mafft-linsi alignment algorithm in MAFFT v7.310, and a maximum likelihood phylogeny was inferred using LG + Γ amino acid substitution model with 100 bootstraps in RAxML version 8.0.26. Bootstrap values below 70 are not shown.

Proteins identified in fraction S3 in *T. immobilis* included both subunits of DNA gyrase (GyrA, GyrB) and three helicases (DnaC, PcrA, and RuvB) that separate the two DNA strands prior to the initiation of replication-repair processes. We also identified in fraction S3 a variety of restriction endonucleases and DNA ligase, LigA, which ligates the DNA following cleavage by endonucleases. Unique to fraction S3 were also proteins in the MutSL mismatch repair and the UvrABCD nucleotide excision repair systems as well as PolA, which insert nucleotides during the repair process. Notably, all four subunits of the UvrABCD complex in *T. immobilis* were exclusively identified in fraction S3, in contrast to the *E. coli* homologs that were exclusively present in fraction S1. The UvrA protein in the *Planctomycetes* is encoded by a fusion of two *uvrA* genes, and thus twice as long as the *E. coli* homolog (Figure 13).

**FIGURE 14:**
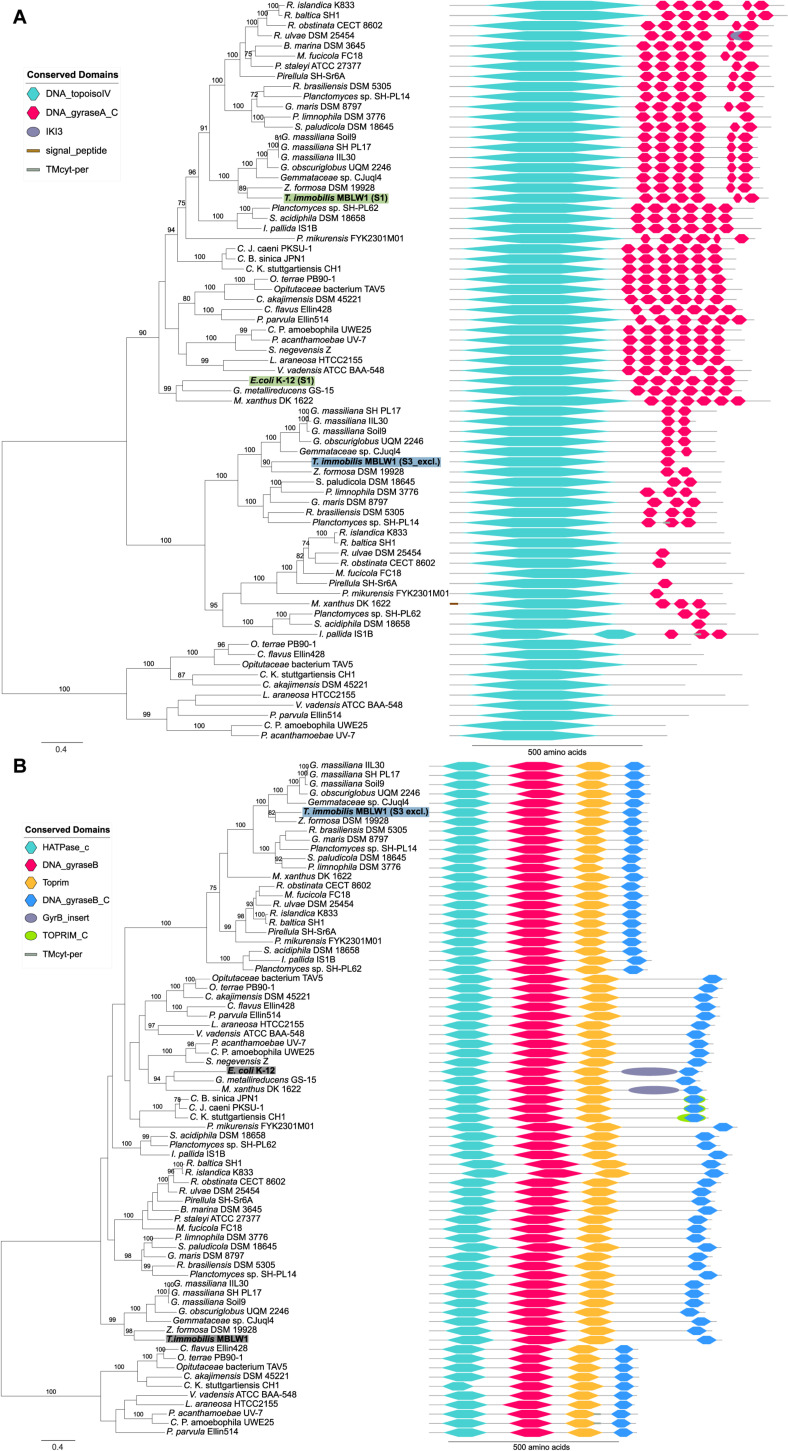
Phylogeny and domain architecture of **(A)** GyrA and **(B)** GyrB in *Planctomycetes* and other bacteria. The species names of the proteins expressed in the subcellular fractionation assay are shown in bold. The corresponding fractions are mentioned in parenthesis and further highlighted by colored boxes [green: Tris (S1), blue: SDS (S3)]. Gray boxes highlight expressed proteins without a clear fractionation profile. Signal peptides and transmembrane domains were annotated using SignalP-5.0b and Phobius 1.01. Conserved domains were assigned to the proteins using the pfam_scan.pl script with a minimum sequence-evalue of 0.01 and the Pfam 32.0 database. The proteins were aligned using the mafft-linsi alignment algorithm in MAFFT v7.310, and a maximum likelihood phylogeny was inferred using LG + Γ amino acid substitution model with 100 bootstraps in RAxML version 8.0.26. Bootstrap values below 70 are not shown.

Interestingly, we identified paralogs to several proteins uniquely expressed in fraction S3 that differed with regard to both domain composition and fractionation patterns. For example, the GyrA and GyrB paralog that clustered with the *E. coli* homolog in the phylogeny were present in both the S1 and the S3 fraction, whereas the S3-exclusive homolog was placed in a distinct clade (Figure 14). The GyrA homologs with a mixed fractionation pattern in *T. immobilis* (GMBLW1_49880) and *E. coli* (b2231) contained six DNA_gyraseA_C domains, while the paralog identified in fraction S3 (GMBLW1_16570) was shorter and only contained 1–3 DNA_gyraseA_C domains (Figure 14A). Likewise, the GyrB protein homolog identified in fraction S3 in *T. immobilis* (GMBLW1_16560) was shorter and lacked an insert between the Toprim and the DNA_gyraseB_C domain present in its paralog (GMBLW1_29410) as well as in the *E. coli* homolog (b3699) (Figure 14B). A previous study identified the S3-exclusive GyrB protein in the paryphoplasmic vesicle membrane fraction (Sagulenko et al., 2017).

For MutS, we identified three paralogous genes in *T. immobilis*, two of which were only found in the S3 fraction, while the third was identified in both fraction S2 and S3. All three MutS proteins in *T. immobilis* contained the MutS_V domain and one of the two proteins identified in the S3 fraction (GMBLW1_32310) contained an identical domain structure to the single MutS protein identified in the S1 fraction in *E. coli* (b2733) (Supplementary Figure 13).

Likewise, the LigA homologs for DNA ligase contained different types and combinations of DNA_ligase domains (Supplementary Figure 14). The *E. coli* homolog (b2411) and the *T. immobilis* homolog with a mixed fractionation pattern (GMBLW1_41300) presented identical domain structures, while the S3-exclusive variant (GMBLW1_22390) was considerably shorter and contained a tryptophan-glycine-arginine rich motif at the C-terminal end (WGR), which is thought to be involved in nucleic acid binding and has been identified in polyA polymerases.

The *T. immobilis* S3-specific proteins also included ribonucleases, such as two paralogs for endo- and exoribonuclease Rbn (GMBLW1_26170, GMBLW1_47640), as well as the 3′-5′exoribonuclease YhaM. The ribonuclease Rbn belongs to the RNase Z protein family and is required for the maturation of tRNA precursors that lack the 3′-terminal CCA sequence, and it also controls 6S RNA, a global transcription regulator. Surprisingly, the *T. immobilis* genome contains as many as three genes for Rbn, two of which were uniquely found in the S3 fraction, while the third was not identified as expressed (GMBLW1_28430). Most bacteria like *E. coli* have only a single copy of this gene, which, however, could not be identified in this experiment.

Among tRNA modifying enzymes in fraction S3 in *T. immobilis*, we identified proteins in the MnmE/MnmG complex that modifies the wobble uridine at position 34 in certain tRNAs, and the MiaAB complex, which methylthiolates the residue at position 37 in tRNAs that read codons beginning with uridine. The MnmG and MiaB proteins were exclusively identified in fraction S3 in *T. immobilis*, while they were exclusively associated with fraction S1 in *E. coli*. There are two paralogus genes for MnmE in *T. immobilis*, both identified in fraction S3 in two of the three replicates. Also, exclusively identified in fraction S1 in *E. coli* and in fraction S3 in *T. immobilis* was peptide deformylase encoded by the *def* gene, which removes the N-terminal fMet after elongation. The RimO protein, which is structurally similar to MiaB but methyltiolates Asp88 in ribosomal protein S8, was exclusively identified in fraction S1 in *E. coli*, while one of the three paralogs was exclusive for fraction S3 in *T. immobilis* (and the other two could not be detected).

Several enzymes that modify rRNA molecules also displayed a remarkable difference between the two species in their fractionation patterns. For example, the 16S rRNA methyltransferase RsmH was exclusively identified in fraction S1 in *E. coli*, while the two RsmH paralogs in *T. immobilis* were solely present in fraction S3, albeit in only one or two replicates. Both proteins in the 23S rRNA pseudoridine synthase RluD/B comples were also solely found in fraction S3 in *T. immobilis*, while identified in fraction S1 in two replicates in *E. coli*.

Finally, we identified the proteins HflX, LepA, RsfS, and RpsZ exclusively in fraction S3 in *T. immobilis*. HflX and RpsZ (ribosomal protein S14) were solely detected in fraction S1 in *E. coli*, while LepA was enriched in fraction S2 and RsfS could not be detected. These proteins have been shown in other bacteria to silence, disassemble and restore ribosomes that have been damaged by heat, high ionic strengths or low temperature. The domain architectures for these proteins are very conserved and the differential fractionation profiles cannot simply be explained by different domain structures. Thus, the enzymes in the translation category uniquely identified in fraction S3 in *T. immobilis* seemed to be mostly involved in the maturation and modification of tRNA and rRNA and in the disassembly of the ribosome.

## Discussion

In this study, we have examined the membrane network of *T. immobilis* using FIB-SEM tomography and analyzed its proteome by LC-MS/MS following a series of protein extractions. The reconstructed *T. immobilis* cells showed the presence of a tunnel-like system made by invaginations of the cytoplasmic membrane, which separated the non-compartmentalized cytoplasmic space from the enlarged periplasmic space. These results are consistent with findings of a continuous cytoplasm with invaginations of the cytoplasmic membrane (Santarella-Mellwig et al., 2013; Acehan et al., 2014; Devos, 2014; Boedeker et al., 2017; Mahajan et al., 2020a), but do not support the hypothesis of a nucleus-like structure in either *T. immobilis* or *G. obscuriglobus* (Lindsay et al., 2001; Fuerst and Sagulenko, 2011).

Our study of the subcellular proteomes of *T. immobilis* (and as a control *Escherichia coli*) by differential solubilization followed by LC-MS/MS analysis identified more than 1,000 proteins in each species. We predicted the subcellular locations of the identified proteins using bioinformatics methods and compared the predictions to the protein extraction patterns. In *E. coli*, the cytoplasmic proteins were mostly recovered in the first Tris-EDTA fraction, inner membrane proteins in the second Triton X-100 fraction and outer membrane proteins in the third SDS-soluble fraction, albeit with some overlap between the fractions. The most dramatic difference between the two species was that about 50% of the cytoplasmic proteins were exclusively identified in the third SDS-soluble fraction in *T. immobilis*, compared to less than 10% in *E. coli*. Below, we discuss these findings in relation to previous interpretations of the cell plan in *G. obscuriglobus* as well as from the broader perspective of features that distinguish prokaryotes from eukaryotes.

A key observation made in this study was that many of the predicted cytoplasmic proteins identified in the SDS-soluble fractions in *T. immobilis* represented recently evolved and highly diverged paralogs of large protein families. This result is related to our previous gene flux analyses, which revealed a massive emergence of new protein families by duplication-divergence in the common ancestor of the *Planctomycetales* as well as in the ancestor of the *Gemmataceae* (Mahajan et al., 2020b). The results presented in this study show that the newly evolved paralogs are expressed and have novel biochemical characteristics, thus providing further support to the importance of paralogization for the evolution of new traits in the *Planctomycete*s (Mahajan et al., 2020b). Paralogization has also been suggested as the driver of the early evolution of the eukaryotic genome, and may well represent a general principle in the evolution toward cellular complexity.

The expanded families in the *Planctomycetes* included serine/threonine kinases (Arcas et al., 2013). The expressed Ser/Thr kinases in *T. immobilis* were identified in the Triton X-100 and SDS soluble fractions and the Pkinase domain(s) were associated with various other domains, which may influence the fractionation patterns. No Ser/Thr kinase proteins were identified in the *E. coli* dataset and hence the larger number of SDS-soluble proteins in the signal transduction category in *T. immobilis* was due to the recovery of proteins uniquely present in this species. The many Ser/Thr kinases are likely to be involved in recently evolved signal transduction pathways in the *Planctomycetes*, just like the Ser/Thr kinase domains are thought to have expanded in the diversifying lineages of the eukaryotes to meet an increased need for new communication pathways.

Paralogs of extracytoplasmic sigma factors (ECFs), which belong to a broad protein family of transcription factors with many young paralogs in the *Gemmataceae* (Jogler et al., 2012; Wiegand et al., 2020) were also identified in the Triton X-100 and SDS-soluble fractions in *T. immobilis*. Furthermore, two paralogs for RNA polymerase recycling factor RapA were identified in the SDS-soluble fraction in *T. immobilis* and these were found to have a different domain structure than the single RapA protein identified in the Tris-EDTA fraction in *E. coli.* In bacteria, the RapA protein competes with the sigma-70 factor for binding to the core RNA polymerase (Jin et al., 2011), whereas the function of the RapA protein in eukaryotes is to make tightly packaged DNA more accessible for RNA polymerase and transcription factors. The new domain structures of the paralogous RapA proteins in *T. immobilis* indicate new or modified functions, and it will be interesting to determine whether these are analogous to those in the eukaryotes.

Likewise, several proteins involved in DNA strand separation and DNA repair processes were exclusively identified in the SDS-soluble fraction in *T. immobilis*, whereas their homologs in *E. coli* were recovered from the Tris-EDTA fraction. The nucleoid in *G. obscuriglobus* is highly condensed with several levels of structural organization (Lieber et al., 2009; Yee et al., 2012) and thus must be uncondensed before DNA repair and transcription can be initiated. Furthermore, it has been hypothesized that transcription occurs at the periphery of the nucleoid, superficially resembling the highly condensed heterochromatin and the transcriptionally active euchromatin of the eukaryotic cell (Yee et al., 2012). Future work is needed to determine the molecular processes involved in DNA repair processes in the *Planctomycetes.*

Yet another category of predicted cytoplasmic proteins solely recovered from the SDS-soluble fraction are involved in the modification and degradation of tRNAs, rRNAs and disassembly of the ribosome. Notably, many novel exonucleases, endonucleases and ribonucleases were inferred as gained in the common ancestor of the *Gemmataceae* in our previous study (Mahajan et al., 2020b). Proteins in the degradosome are membrane-associated in *E. coli* and the most recent hypothesis is that RNA molecules are transcribed in the nucleoid and translated in the cytoplasm, but processed and degraded near the inner membranes (Moffitt et al., 2016; Kannaiah et al., 2019). In *T. immobilis*, a potential role for these enzymes in the degradation of imported molecules should also be considered, which takes place in the periplasmic space and maybe even inside vacuoles, as in the eukaryotes. Thus, some enzymes in the molecular recycling processes may not be soluble in Tris-EDTA because they are associated with the membrane and/or embedded inside vacuoles.

The endocellular membranes in the eukaryotic cell serves as a transportation system, enabling different cellular processes to occur in distinct parts of the cell. The elaborate intracellular membrane system in the *Planctomycetes* could potentially also enable some separation of cellular processes in time and space, by forming flexibly arranged tunnels and caves. One hypothesis is that protein complexes involved in DNA replication and repair, and in the synthesis and degradation of transcription and translation components are spatially localized to different segments of these tunnels. The RNA and protein molecules may then diffuse or be transported along the tunnels, and encounter various “molecular workstations,” depending on their molecular tags and stages in the recycling process. Such potential caves and tunnels formed by the intracellular membrane networks might represent a pre-stage to the evolution of the membrane-enclosed structures in the eukaryotic cell.

We also considered the possibility that the presence of a nuclear-like membrane could help to separate transcription, protein synthesis and the degradation of translational component, much like in the eukaryotic cell. However, our data did not support the hypothesis of a nuclear compartment, which raises questions about the identity of the pore-containing membrane in *G. obscuriglobus* that was thought to correspond to the nuclear membrane (Sagulenko et al., 2017). A mass-spectrometry analysis showed that this membrane layer contained beta barrel proteins, lipoproteins and proteins with cell surface motifs, homologs to which were uniquely identified in the SDS-soluble fraction in *T. immobilis*. We also observed pores in previous cell wall preparations of *T. immobilis* (Mahajan et al., 2020a). We therefore favor the hypothesis that the pore-containing membrane corresponds to the outer membrane, an interpretation that has also been suggested by others (Jogler et al., 2019).

Our findings underscore the importance of paralogization and domain shuffling as a mechanism for functional innovation and adaptation to a more complex cell structure in the *Planctomycetes* (Mahajan et al., 2020b). We hypothesize that an open spatial organization of molecular processes represents an intermediate stage in the transition toward a fully compartmentalized cell. The observed fractionation patterns are admittedly very complex and we can think of several explanations for the findings. Some proteins may for example interact with new molecular complexes, while others may be embedded inside vesicles or be attached to the intracellular membranes. More detailed studies of each individual protein in protein families with many paralogs will be needed to determine the link between new domain composition patterns and new functions. Future experimental research should specifically focus on the temporal and spatial organization of systems related to signal transduction, transcription regulation, DNA repair and RNA recycling to test the hypotheses proposed in this article.

## Data Availability Statement

FIB-SEM image stacks and IMOD segmentations are deposited to the EMPIAR EM Public Image Archive (EMPIAR-10554: *T. immobilis*, EMPIAR-10553: *G. obscuriglobus*). The mass spectrometry proteomics data have been deposited to the ProteomeXchange Consortium via the PRIDE (Perez-Riverol et al., 2019) partner repository with the dataset identifier PXD022526 (*E. coli*) and PXD022559 (*T. immobilis*). Transmission Electron Microscopy micrographs of subcellular fractions, movies from FIB-SEM segmentations and 3D-reconstructions are deposited to the BioStudies database.

## Author Contributions

CS, KD, and SA designed the study and analyzed the data and wrote the manuscript. CS and SL performed bacterial cultivation, protein extraction and proteomics experiments. CS performed the reconstruction of the FIB-SEM data. KD identified the sequence logos and the signal peptide motif, performed the analyses of the large-scale proteomics data including subcellular localization predictions, estimates of relative protein abundance and comparison to proteins identified previously in cell wall preparations and membrane layers. MM and AO performed the phylogenetic analyses. KD and MM analyzed the pilins and SBP_bac_10 domain proteins. All authors have read and approved the submitted manuscripts.

## Conflict of Interest

The authors declare that the research was conducted in the absence of any commercial or financial relationships that could be construed as a potential conflict of interest.

## References

[B1] Acehan D. Santarella-Mellwig R. Devos D. P. (2014). Abacterial tubulovesicular network *J. Cell Sci.* 127 277–280. 24259664 10.1242/jcs.137596

[B2] Almagro Armenteros J. J. Tsirigos K. D. Sønderby C. K. Petersen T. N. Winther O. Brunak S. (2019). SignalP 5.0 improves signal peptide predictions using deep neural networks. *Nat. Biotechnol.* 37 420–423. 10.1038/s41587-019-0036-z 30778233

[B3] Altschul S. F. Madden T. L. Schäffer A. A. Zhang J. Zhang Z. Miller W. (1997). Gapped BLAST and PSI-BLAST: a new generation of protein database search programs. *Nucleic Acids Res.* 25 3389–3402. 10.1093/nar/25.17.3389 9254694 PMC146917

[B4] Altschul S. F. Wootton J. C. Gertz E. M. Agarwala R. Morgulis A. Schäffer A. A. (2005). Protein database searches using compositionally adjusted substitution matrices. *FEBS J.* 272 5101–5109. 10.1111/j.1742-4658.2005.04945.x 16218944 PMC1343503

[B5] Arcas A. Cases I. Rojas A. M. (2013). Serine/threonine kinases and E2-ubiquitin conjugating enzymes in Planctomycetes: unexpected findings. *Antonie Van Leeuwenhoek* 104 509–520. 10.1007/s10482-013-9993-2 23918348

[B6] Babu M. M. Priya M. L. Selvan A. T. Madera M. Gough J. Aravind L. (2006). A database of bacterial lipoproteins (DOLOP) with functional assignments to predicted lipoproteins. *J. Bacteriol.* 188 2761–2773. 10.1128/jb.188.8.2761-2773.2006 16585737 PMC1446993

[B7] Berven F. S. Flikka K. Jensen H. B. Eidhammer I. (2004). BOMP: a program to predict integral beta-barrel outer membrane proteins encoded within genomes of Gram-negative bacteria. *Nucleic Acids Res.* 32 W394–W399. 15215418 10.1093/nar/gkh351 PMC441489

[B8] Boedeker C. Schüler M. Reintjes G. Jeske O. van Teeseling M. C. F. Jogler M. (2017). Determining the bacterial cell biology of Planctomycetes. *Nat. Commun.* 8:14853. 10.1038/ncomms14853 PMC5394234 28393831

[B9] Capella-Gutiérrez S. Silla-Martínez J. M. Gabaldón T. (2009). trimAl: a tool for automated alignment trimming in large-scale phylogenetic analyses. *Bioinformatics* 25 1972–1973. 10.1093/bioinformatics/btp348 19505945 PMC2712344

[B10] Cox J. Mann M. (2008). MaxQuant enables high peptide identification rates, individualized p.p.b.-range mass accuracies and proteome-wide protein quantification. *Nat. Biotechnol.* 26 1367–1372. 10.1038/nbt.1511 19029910

[B11] Cox J. Neuhauser N. Michalski A. Scheltema R. A. Olsen J. V. Mann M. (2011). Andromeda: a peptide search engine integrated into the MaxQuant environment. *J. Proteome Res.* 10 1794–1805. 10.1021/pr101065j 21254760

[B12] Devos D. P. (2014). PVC bacteria: variation of, but not exception to, the Gram-negative cell plan. *Trends Microbiol.* 22 14–20. 10.1016/j.tim.2013.10.008 24286661

[B13] Eddy S. R. (2009). A new generation of homology search tools based on probabilistic inference. *Genome Inform.* 23 205–211. 20180275

[B14] Forterre P. Gribaldo S. (2010). Bacteria with a eukaryotic touch: a glimpse of ancient evolution? *Proc. Natl. Acad. Sci. U S A.* 107 12739–12740. 10.1073/pnas.1007720107 20624972 PMC2919924

[B15] Fuerst J. A. Sagulenko E. (2011). Beyond the bacterium: Planctomycetes challenge our concepts of microbial structure and function. *Nat. Rev. Microbiol.* 9 403–413. 10.1038/nrmicro2578 21572457

[B16] Fuerst J. A. Webb R. I. (1991). Membrane-bounded nucleoid in the eubacterium *Gemmata obscuriglobus*. *Proc. Natl. Acad. Sci. U.S.A.* 88 8184–8188. 10.1073/pnas.88.18.8184 11607213 PMC52471

[B17] Hieu C. X. Voigt B. Albrecht D. Becher D. Lombardot T. Glöckner F. O. (2008). Detailed proteome analysis of growing cells of the planctomycete *Rhodopirellula baltica* SH1T. *Proteomics* 8 1608–1623. 10.1002/pmic.200701017 18340632

[B18] Hoang D. T. Chernomor O. von Haeseler A. Minh B. Q. Vinh L. S. (2018). UFBoot2: improving the ultrafast bootstrap approximation. *Mol. Biol. Evol.* 35 518–522. 10.1093/molbev/msx281 29077904 PMC5850222

[B19] Iudin A. Korir P. K. Salavert-Torres J. Kleywegt G. J. Patwardhan A. (2016). EMPIAR: a public archive for raw electron microscopy image data. *Nat. Methods* 13 387–388. 10.1038/nmeth.3806 27067018

[B20] Jin D. J. Zhou Y. N. Shaw G. Ji X. (2011). Structure and function of RapA: a bacterial Swi2/Snf2 protein required for RNA polymerase recycling in transcription. *Biochim. Biophys. Acta* 1809 470–475. 10.1016/j.bbagrm.2011.03.003 21419241 PMC3142277

[B21] Jogler C. Waldmann J. Huang X. Jogler M. Glöckner F. O. Mascher T. (2012). Identification of proteins likely to be involved in morphogenesis, cell division, and signal transduction in planctomycetes by comparative genomics. *J. Bacteriol.* 194 6419–6430. 10.1128/jb.01325-12 23002222 PMC3497475

[B22] Jogler C. Wiegand S. Devos D. P. (2019). Commentary: manifold routes to a nucleus. *Front. Microbiol.* 10:1198. 10.3389/fmicb.2019.01198 31214141 PMC6554331

[B23] Juncker A. S. Willenbrock H. von Heijne G. Brunak S. Nielsen H. Krogh A. (2003). Prediction of lipoprotein signal peptides in gram negative bacteria. *Protein Sci.* 12 1652–1662. 10.1110/ps.0303703 12876315 PMC2323952

[B24] Käll L. Krogh A. Sonnhammer E. L. L. (2004). A combined transmembrane topology and signal peptide prediction method. *J. Mol. Biol.* 338 1027–1036. 10.1016/j.jmb.2004.03.016 15111065

[B25] Käll L. Krogh A. Sonnhammer E. L. L. (2007). Advantages of combined transmembrane topology and signal peptide prediction-the Phobius web server. *Nucleic Acids Res.* 35 429–432. 10.1093/nar/gkm256 PMC1933244 17483518

[B26] Kannaiah S. Livny J. Amster-Choder O. (2019). Spatiotemporal organization of the *E. coli* transcriptome: translation independence and engagement in regulation. *Mol. Cell* 76 574–589.e7. 31540875 10.1016/j.molcel.2019.08.013

[B27] Katoh K. Kuma K. Toh H. Miyata T. (2005). MAFFT version 5: improvement in accuracy of multiple sequence alignment. *Nucleic Acids Res.* 33 511–518. 10.1093/nar/gki198 15661851 PMC548345

[B28] Katoh K. Standley D. M. (2013). MAFFT multiple sequence alignment software version 7: improvements in performance and usability. *Mol. Biol. Evol.* 30 772–780. 10.1093/molbev/mst010 23329690 PMC3603318

[B29] König E. Schlesner H. Hirsch P. (1984). Cell wall studies on budding bacteria of the Planctomyces/Pasteuria group and on a *Prosthecomicrobium sp*. *Arch. Microbiol.* 138 200–205. 10.1007/bf00402120

[B30] Krogh A. Larsson B. von Heijne G. Sonnhammer E. L. (2001). Predicting transmembrane protein topology with a hidden markov model: application to complete genomes. *J. Mol. Biol.* 305 567–580. 10.1006/jmbi.2000.4315 11152613

[B31] Kulichevskaya I. S. Ivanova A. A. Baulina O. I. Rijpstra W. I. C. Damst J. S. S. (2017). Fimbriiglobus ruber gen. nov., sp. nov., a Gemmata-like planctomycete from *Sphagnum peat* bog and the proposal of *Gemmataceae fam*. nov. *Int. J. Syst. Evol. Microbiol.* 67 218–224. 10.1099/ijsem.0.001598 27902209

[B32] Lee K.-C. Webb R. I. Fuerst J. A. (2009). The cell cycle of the planctomycete *Gemmata obscuriglobus* with respect to cell compartmentalization. *BMC Cell Biol.* 10:4. 10.1186/1471-2121-10-4 19144151 PMC2656463

[B33] Lieber A. Leis A. Kushmaro A. Minsky A. Medalia O. (2009). Chromatin organization and radio resistance in the bacterium *Gemmata obscuriglobus*. *J. Bacteriol.* 191 1439–1445. 10.1128/jb.01513-08 19074379 PMC2648202

[B34] Liesack W. König H. Schlesner H. Hirsch P. (1986). Chemical composition of the peptidoglycan-free cell envelopes of budding bacteria of the Pirella/Planctomyces group. *Arch. Microbiol.* 145 361–366. 10.1007/bf00470872

[B35] Lindsay M. R. Webb R. I. Strous M. Jetten M. S. M. Butler M. K. Forde R. J. (2001). Cell compartmentalisation in Planctomycetes: novel types of structural organisation for the bacterial cell. *Arch. Microbiol.* 175 413–429. 10.1007/s002030100280 11491082

[B36] Mahajan M. Seeger C. Yee B. Andersson S. G. E. (2020a). Evolutionary remodelling of the cell envelope in bacteria of the Planctomycetes phylum. *Genome Biol. Evol.* 12 1528–1548. 10.1093/gbe/evaa159 32761170 PMC7533069

[B37] Mahajan M. Yee B. Hägglund E. Guy L. Fuerst J. A. Andersson S. G. E. (2020b). Paralogization and new protein architectures in Planctomycetes bacteria with complex cell structures. *Mol. Biol. Evol.* 37 1020–1040. 10.1093/molbev/msz287 31808939

[B38] Mcinerney J. O. Martin W. F. Koonin E. V. Allen J. F. Galperin M. Y. Lane N. (2011). Planctomycetes and eukaryotes: a case of analogy not homology. *BioEssays* 33 810–817. 10.1002/bies.201100045 21858844 PMC3795523

[B39] Minh B. Q. Schmidt H. A. Chernomor O. Schrempf D. Woodhams M. D. von Haeseler A. (2020). IQ-TREE 2: new models and efficient methods for phylogenetic inference in the genomic era. *Mol. Biol. Evol.* 37 1530–1534. 10.1093/molbev/msaa015 32011700 PMC7182206

[B40] Moffitt J. R. Pandey S. Boettiger A. N. Wang S. Zhuang X. (2016). Spatial organization shapes the turnover of a bacterial transcriptome. *eLife* 5:e13065. 10.7554/eLife.13065 PMC4874777 27198188

[B41] Nakayama H. Kurokawa K. Lee B. L. (2012). Lipoproteins in bacteria: structures and biosynthetic pathways. *FEBS J.* 279 4247–4268. 10.1111/febs.12041 23094979

[B42] Neumann S. Wessels H. J. C. T. Rijpstra W. I. C. Sinninghe Damsté J. S. Kartal B. Jetten M. S. M. (2014). Isolation and characterization of a prokaryotic cell organelle from the anammox bacterium *Kuenenia stuttgartiensis*. *Mol. Microbiol.* 94 794–802. 10.1111/mmi.12816 25287816

[B43] Perez-Riverol Y. Csordas A. Bai J. Bernal-Llinares M. Hewapathirana S. Kundu D. J. (2019). The PRIDE database and related tools and resources in 2019: improving support for quantification data. *Nucl. Acids Res.* 47 D442–D450. 10.1093/nar/gky1106 30395289 PMC6323896

[B44] Punta M. Coggill P. C. Eberhardt R. Y. Mistry J. Tate J. Boursnell C. (2012). The Pfam protein families database. *Nucleic Acids Res.* 40 D290–D301. 22127870 10.1093/nar/gkr1065 PMC3245129

[B45] Sagulenko E. Morgan G. P. Webb R. I. Yee B. Lee K. C. Fuerst J. A. (2014). Structural studies of Planctomycete *Gemmata obscuriglobus* support cell compartmentalisation in a bacterium. *PLoS One* 9:e91344. 10.1371/journal.pone.0091344 24632833 PMC3954628

[B46] Sagulenko E. Nouwens A. Webb R. I. Green K. Yee B. Morgan G. (2017). Nuclear pore-like structures in a compartmentalized bacterium. *PLoS One* 12:e0169432. 10.1371/journal.pone.0169432 28146565 PMC5287468

[B47] Santarella-Mellwig R. Pruggnaller S. Roos N. Mattaj I. W. Devos D. P. (2013). Three-dimensional reconstruction of bacteria with a complex endomembrane system. *PLoS Biol.* 11:e1001565. 10.1371/journal.pbio.1001565 23700385 PMC3660258

[B48] Schnaitman C. A. (1970). Protein composition of the cell wall and cytoplasmic membrane of *Escherichia coli*. *J. Bacteriol.* 104 890–901. 10.1128/jb.104.2.890-901.1970 4099097 PMC285073

[B49] Schnaitman C. A. (1971). Solubilization of the cytoplasmic membrane of *Escherichia coli* by triton X-100. *J. Bacteriol.* 108 545–552. 10.1128/jb.108.1.545-552.1971 4941569 PMC247096

[B50] Seeger C. Butler M. K. Yee B. Mahajan M. Fuerst J. A. Andersson S. G. E. (2017). Tuwongella immobilis gen. nov., sp. nov., a novel non-motile bacterium within the phylum Planctomycetes. *Int. J. Syst. Evol. Microbiol.* 67 4923–4929. 10.1099/ijsem.0.002271 29087267 PMC5845749

[B51] Seemann T. (2014). Prokka: rapid prokaryotic genome annotation. *Bioinformatics* 30 2068–2069. 10.1093/bioinformatics/btu153 24642063

[B52] Sonnhammer E. L. von Heijne G. Krogh A. (1998). A hidden markov model for predicting transmembrane helices in protein sequences. *Proc. Int. Conf. Intell. Syst. Mol. Biol*. 6 175–182. 9783223

[B53] Stamatakis A. (2014). RAxML version 8: a tool for phylogenetic analysis and post-analysis of large phylogenies. *Bioinformatics* 30 1312–1313. 10.1093/bioinformatics/btu033 24451623 PMC3998144

[B54] Stöver B. C. Müller K. F. (2010). TreeGraph 2: combining and visualizing evidence from different phylogenetic analyses. *BMC Bioinformatics* 11:7. 10.1186/1471-2105-11-7 20051126 PMC2806359

[B55] Studholme D. J. Fuerst J. A. Bateman A. (2004). Novel protein domains and motifs in the marine planctomycete *Rhodopirellula baltica*. *FEMS Microbiol. Lett.* 236 333–340. 10.1111/j.1574-6968.2004.tb09666.x 15251216

[B56] Tatusov R. L. Galperin M. Y. Natale D. A. Koonin E. V. (2000). The COG database: a tool for genome-scale analysis of protein functions and evolution. *Nucleic Acids Res.* 28 33–36. 10.1093/nar/28.1.33 10592175 PMC102395

[B57] van Teeseling M. C. F. Mesman R. J. Kuru E. Espaillat A. Cava F. Brun Y. V. (2015). Anammox Planctomycetes have a peptidoglycan cell wall. *Nat. Commun.* 6:6878. 10.1038/ncomms7878 PMC4432595 25962786

[B58] Wiegand S. Jogler M. Boedeker C. Pinto D. Vollmers J. Rivas-Marín E. (2020). Cultivation and functional characterization of 79 Planctomycetes uncovers their unique biology. *Nat. Microbiol.* 5 126–140. 31740763 10.1038/s41564-019-0588-1 PMC7286433

[B59] Wiegand S. Jogler M. Jogler C. (2018). On the maverick Planctomycetes. *FEMS Microbiol. Rev.* 42 739–760. 10.1093/femsre/fuy029 30052954

[B60] Yee B. Sagulenko E. Morgan G. P. Webb R. I. Fuerst J. A. (2012). Electron tomography of the nucleoid of Gemmata obscuriglobus reveals complex liquid crystalline cholesteric structure. *Front. Microbiol.* 3:326. 10.3389/fmicb.2012.00326 22993511 PMC3440768

[B61] Yu N. Y. Wagner J. R. Laird M. R. Melli G. Rey S. Lo R. (2010). PSORTb 3.0: improved protein subcellular localization prediction with refined localization subcategories and predictive capabilities for all prokaryotes. *Bioinformatics* 26 1608–1615. 10.1093/bioinformatics/btq249 20472543 PMC2887053

