# Supplementary material for: The Subcellular Proteome of a Planctomycetes Bacterium Shows That Newly Evolved Proteins Have Distinct Fractionation Patterns
Source: Front Microbiol. 2021 May 4;12:643045. doi: 10.3389/fmicb.2021.643045 (PMC8567305; doi:10.3389/fmicb.2021.643045)
Supplement: Supplementary file 1 [file Data_Sheet_1.docx]

***Supplementary Figures***


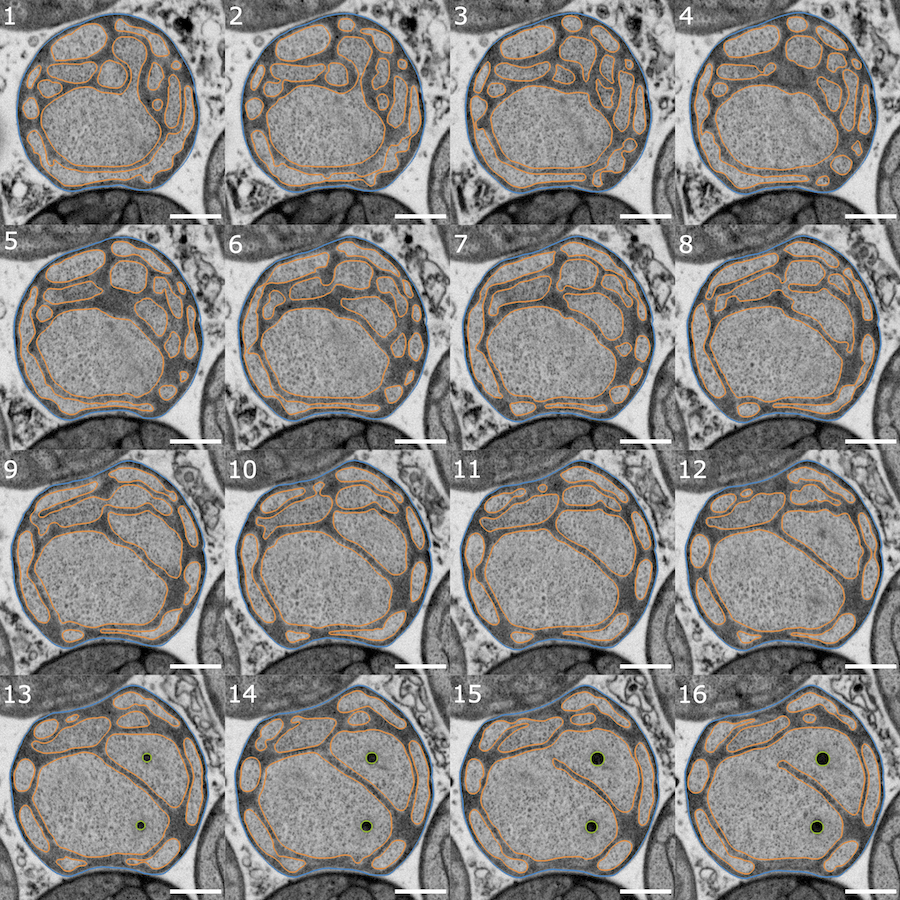


**Supplementary Figure S1.** Illustration of segmentation of *T. immobilis* cell “C3”. Contours were manually drawn (blue: cell envelope; orange: cytoplasmic membrane; green: polyphosphate granules). Segmented slices and the 3D-reconstruction are shown in Supplementary Movie 1. Scale bar: 500 nm.


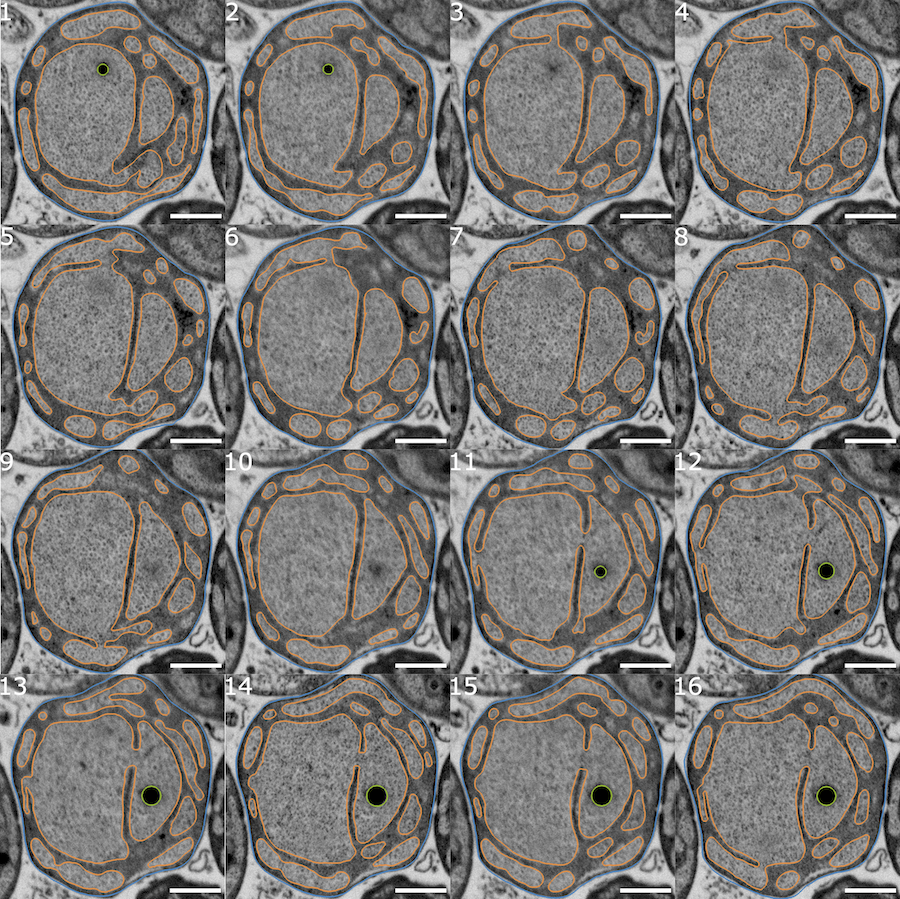


**Supplementary Figure S2.** Illustration of segmentation of *T. immobilis* cell “C4”. Contours were manually drawn (blue: cell envelope; orange: cytoplasmic membrane; green: polyphosphate granules). Segmented slices and the 3D-reconstruction are shown in Supplementary Movie 2. Scale bar: 500 nm.


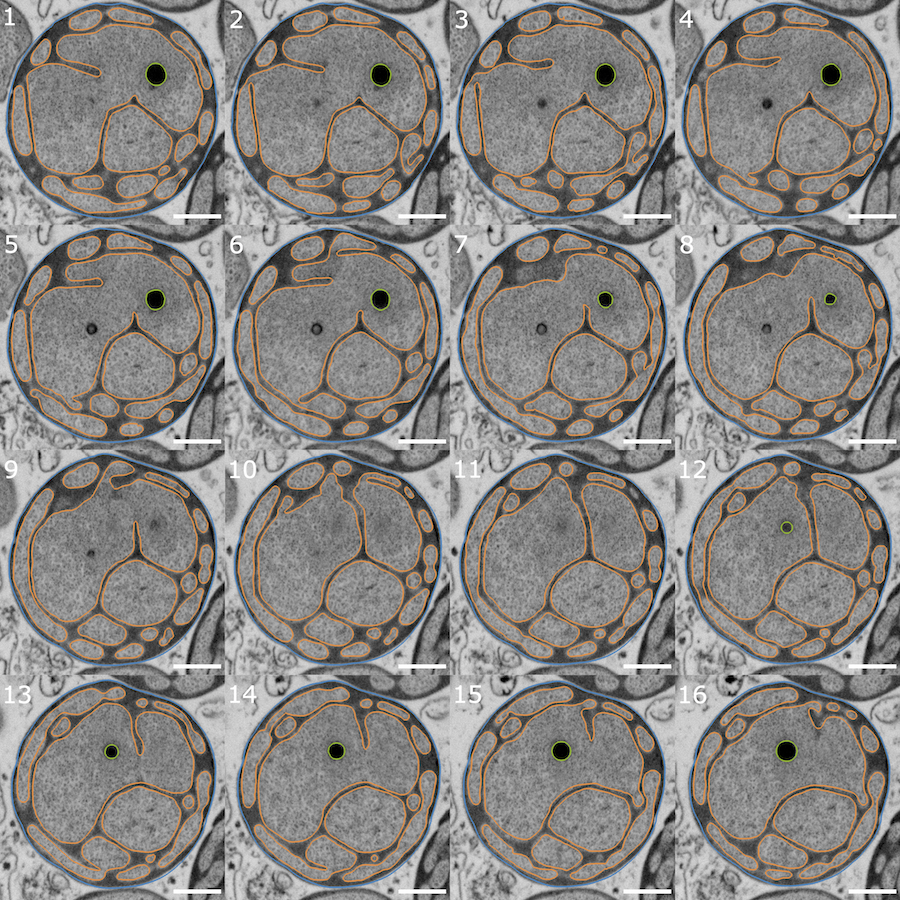


**Supplementary Figure S3.** Illustration of segmentation of *T. immobilis* cell “C5”. Contours were manually drawn (blue: cell envelope; orange: cytoplasmic membrane; green: polyphosphate granules). Segmented slices and the 3D-reconstruction are shown in Supplementary Movie 3. Scale bar: 500 nm.


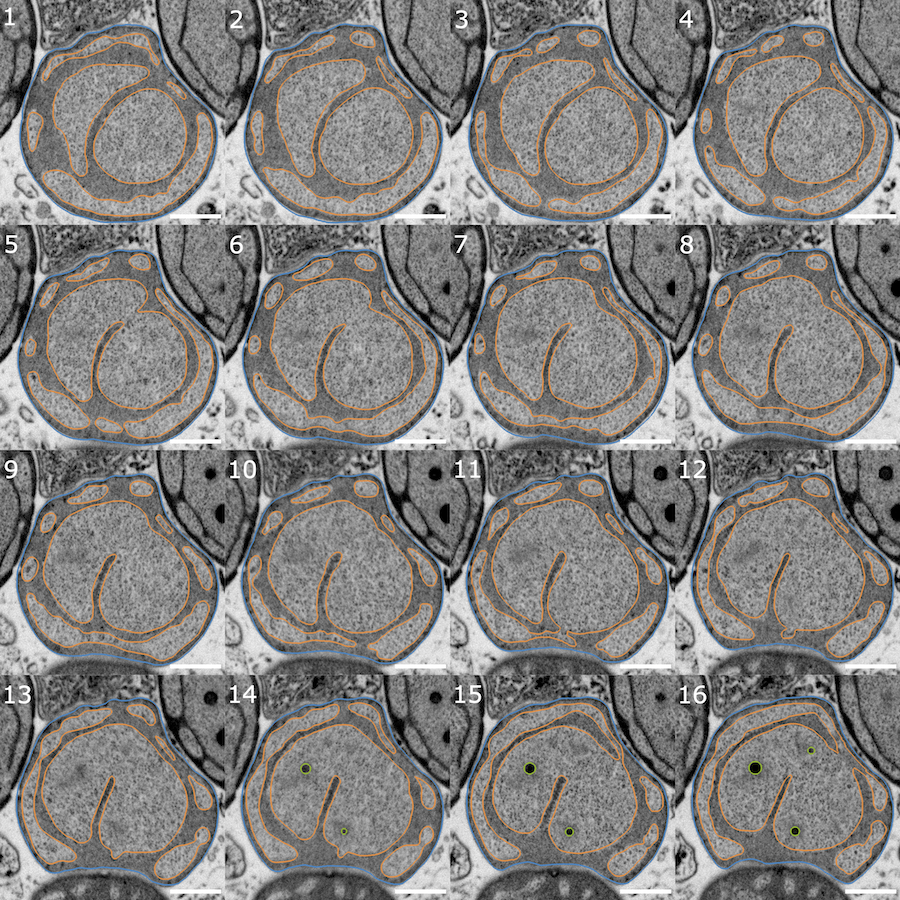


**Supplementary Figure S4.** Illustration of segmentation of *T. immobilis* cell “C6”. Contours were manually drawn (blue: cell envelope; orange: cytoplasmic membrane; green: polyphosphate granules). Segmented slices and the 3D-reconstruction are shown in Supplementary Movie 4. Scale bar: 500 nm.


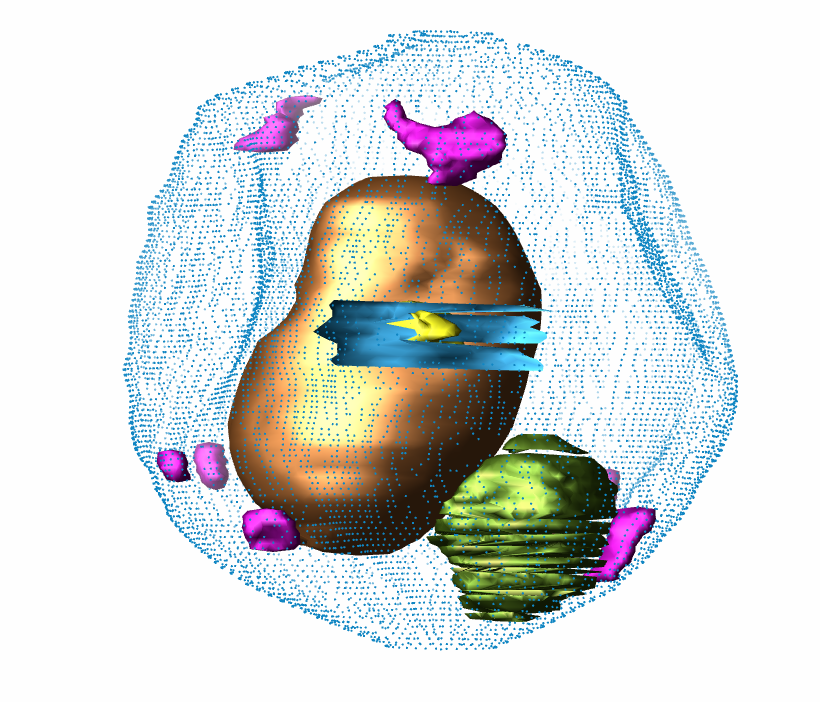


**Supplementary Figure S5.** Partially reconstructed cell displaying signs of membrane damage and lysis (blue dotted contour: cell envelope; blue solid mesh: membrane hole; pink mesh: empty membrane vesicles; green: ruptured membrane vesicle). The 3D-reconstruction is shown in Supplementary Movie 5.


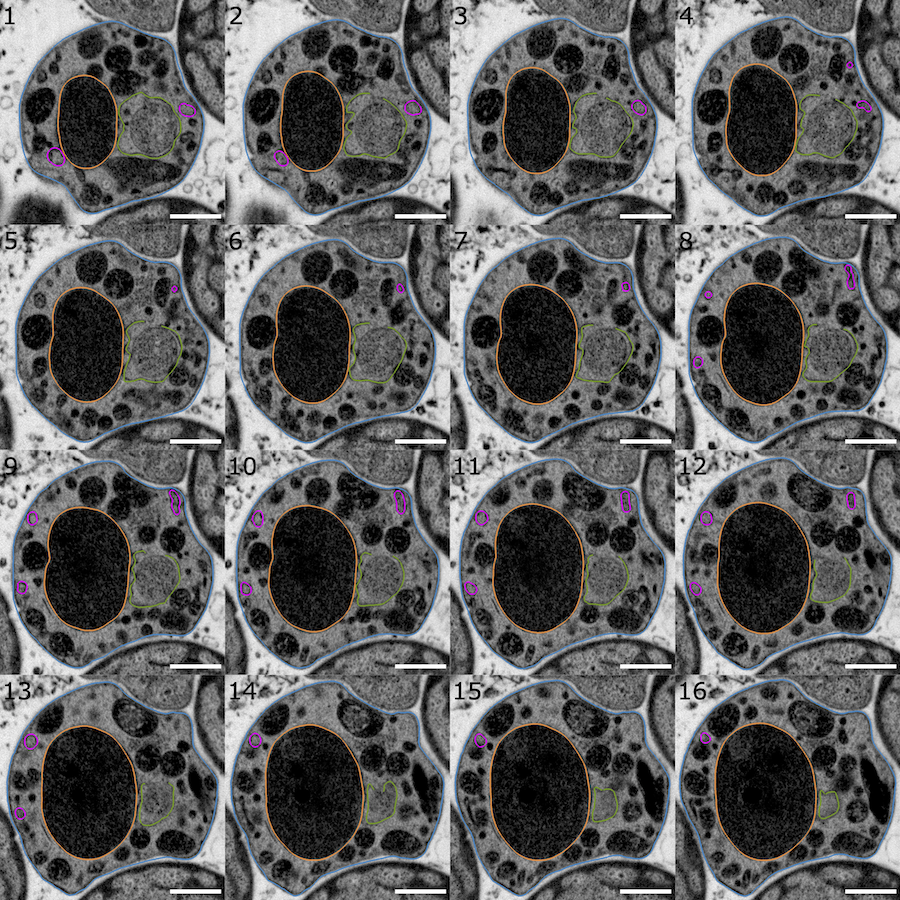


**Supplementary Figure S6.** Illustration of segmentation of *T. immobilis* cell displaying membrane damage and other signs of cell lysis. Contours were manually drawn (blue: cell envelope; orange: intact cytoplasmic membrane vesicle; green: ruptured cytoplasmic membrane vesicle). Segmented slices and the 3D-reconstruction are shown in Supplementary Movie 5. Scale bar: 500 nm.


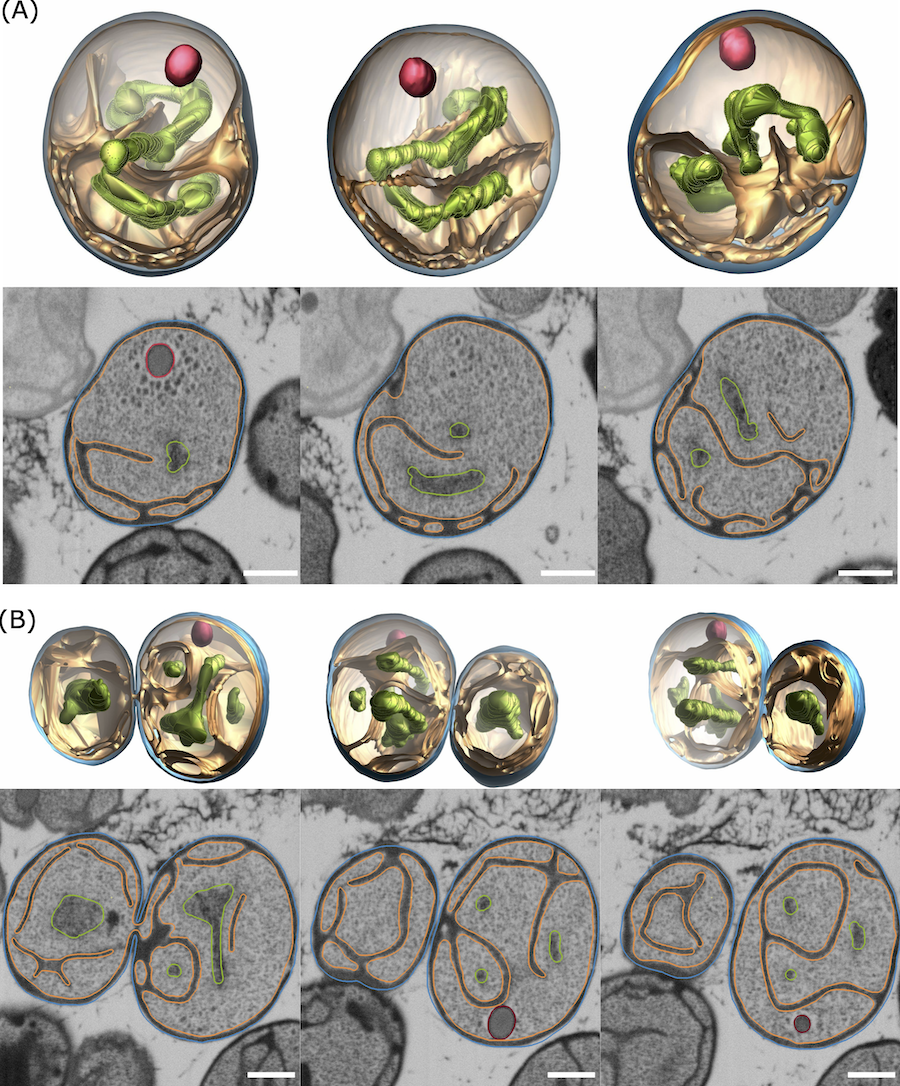


**Supplementary Figure S7.** 3D-reconstruction of *Gemmata obscuriglobus* based on FIB-SEM analysis. Snapshots of 3D-model (top) and segmented FIB-SEM micrographs (bottom) of single (**A**) and budding cell (**B**). Coloured structures correspond to the cytoplasmic membrane (orange), cell envelope (blue), nucleoid (green) and a large spherical particle (red). Scale bar: 500 nm.

**Supplementary Figure S8.** Batch cultivation of (A) *T. immobilis* and (B) *E. coli* K12 MG1655*.* Average and standard deviation of OD_600_ values from three biological replicates are shown as a function of time. A logistic growth model was fitted to the experimental data using GraphPad Prism to estimate the generation times (g_T.immobilis_ = 8h, g_E.coli_ = 35 min).

**Supplementary Figure S9.** Analysis of *E. coli* subcellular fractions (S1, S2, S3, biological triplicates) by SDS-PAGE. M: marker with corresponding molecular weights in kDa.

**Supplementary Figure S10.** Subcellular fractionation profiles and domain architectures of protein groups in *T. immobilis* and *E. coli* with N_methyl prepilin cleavage motif or type I/II signal peptides. The heatmap shows the relative abundance of the experimentally identified proteins as a function of the respective subcellular fraction (Tris soluble (S1), Tris/TX-100 soluble (S2), and Tris/SDS soluble (S3)).

**Supplementary Figure S11.** Phylogenetic analysis of GMBLW1_25620 (WP_162656607.1) from *T. immobilis* and homologous proteins from *Planctomycetales* species. GMBLW1_25620 is a predicted lipoprotein which was exclusively identified in the SDS-soluble fraction (S3) in *T. immobilis* and the third highest scoring protein in the cell wall proteome of Mahajan et al. 2020a. Branches with a bootstrap (UFBoot) support greater than 95% are indicated by black dots. The protein fasta file with the corresponding sequences is deposited at the Biostudies archive (S12_phylogeny_proteins_GMBLW1_25620.fasta).

**Supplementary Figure S12.** Phylogeny and domain architecture of RapA in *Planctomycetes* and other bacteria. The species names of the proteins expressed in the subcellular fractionation assay are shown in bold, and the fraction in which the proteins were expressed in mentioned in brackets next to the species name. Signal peptides and transmembrane domains were annotated using SignalP-5.0b and Phobius 1.01. Conserved domains were assigned to the proteins using the pfam_scan.pl script with a minimum sequence-evalue of 0.01 and the Pfam 32.0 database. The proteins were aligned using the mafft-linsi alignment algorithm in MAFFT v7.310, and a maximum likelihood phylogeny was inferred using LG + Γ amino acid substitution model with 100 bootstraps in RAxML version 8.0.26. Bootstrap values below 70 are not shown. The alignments and protein IDs related to the respective species are deposited at Biostudies (*Submission in progress*).

**Supplementary Figure S13.** Phylogeny and domain architecture of MutS in *Planctomycetes* and other bacteria. The species names of the proteins expressed in the subcellular fractionation assay are shown in bold, and the fraction in which the proteins were expressed in mentioned in brackets next to the species name. Signal peptides and transmembrane domains were annotated using SignalP-5.0b and Phobius 1.01. Conserved domains were assigned to the proteins using the pfam_scan.pl script with a minimum sequence-evalue of 0.01 and the Pfam 32.0 database. The proteins were aligned using the mafft-linsi alignment algorithm in MAFFT v7.310, and a maximum likelihood phylogeny was inferred using LG + Γ amino acid substitution model with 100 bootstraps in RAxML version 8.0.26. Bootstrap values below 70 are not shown. The alignments and protein IDs related to the respective species are deposited at Biostudies (*Submission in progress*).

**Supplementary Figure S14.** Phylogeny and domain architecture of LigA in *Planctomycetes* and other bacteria. The species names of the proteins expressed in the subcellular fractionation assay are shown in bold, and the fraction in which the proteins were expressed in mentioned in brackets next to the species name. Signal peptides and transmembrane domains were annotated using SignalP-5.0b and Phobius 1.01. Conserved domains were assigned to the proteins using the pfam_scan.pl script with a minimum sequence-evalue of 0.01 and the Pfam 32.0 database. The proteins were aligned using the mafft-linsi alignment algorithm in MAFFT v7.310, and a maximum likelihood phylogeny was inferred using LG + Γ amino acid substitution model with 100 bootstraps in RAxML version 8.0.26. Bootstrap values below 70 are not shown. The alignments and protein IDs related to the respective species are deposited at Biostudies (*Submission in progress*).
