# Supplementary material for: The Subcellular Proteome of a Planctomycetes Bacterium Shows That Newly Evolved Proteins Have Distinct Fractionation Patterns
Source: Front Microbiol. 2021 May 4;12:643045. doi: 10.3389/fmicb.2021.643045 (PMC8567305; doi:10.3389/fmicb.2021.643045)
Supplement: Supplementary file 2 [file Table_1.docx]

***Supplementary Material***

**Legends to Supplementary Tables**

**Supplementary Table S1.** Summary of IMOD segmentations of FIB-SEM generated volumes of *Tuwongella immobilis* and *Gemmata obscuriglobus.*

**Supplementary Table S2.** Protein yields of *T. immobilis* subcellular fractions. S0: Total cell lysate; S1: Tris soluble; S2: Tris/TX-100 soluble; S3: Tris/SDS soluble.

**Supplementary Table S3.** Comprehensive summary of LC-MS/MS results for *T. immobilis* and *E. coli*. LFQ-intensities are listed for each identified protein and replicate of the three subcellular fractions (LFQ_S1_1–3, LFQ_S2_1–3, LFQ_S3_1–3). Predictions are included for subcellular localization (PSORTb), transmembrane domains (Phobius), lipoproteins (LipoP), outer membrane proteins (BOMP). Signal peptides were predicted by SignalP-5.0. Functional predictions are based on COG categories. Predictions of protein domains are based on Pfam. Both short names and product names are based on PROKKA. The presence of a *Planctomycetes* specific signal peptide (“cell_surface_motif”) is indicated for the respective proteins. Proteins containing the SBP_bac_10 domain are highlighted. The table contains the complete species proteome (GenBank: LR593887.1 for *T. immobilis*, GenBank: U00096.3 for *E. coli*) including both identified and non-identified proteins.

**Supplementary Table S4.** Proteomes of *T. immobilis* (1569 proteins) and *E. coli* (1233 proteins) identified by subcellular fractionation and LC-MS/MS analysis.

**Supplementary Table S5.** Predicted subcellular localization by PSORTb of the *T. immobilis* and *E. coli* subcellular proteomes.

**Supplementary Table S6.** Predictions of subcellular localizations for the subcellular proteomes of *T. immobilis* and *E. coli*. Lipoproteins predicted by LipoP and proteins with predicted beta barrels by BOMP are included in the table even if the PSORTb prediction is unknown.

**Supplementary Table S7.** Summary of 198 experimentally identified proteins related to the COG category M (Cell wall/membrane/envelop biogenesis) in *T. immobilis* and *E. coli*.

**Supplementary Table S8.** Summary of proteins in *T. immobilis* and *E. coli* with with predicted N_methyl, SBP_bac domains (_10, _3/5/6/8/11) or signal peptides. Experimentally identified proteins are summarised together with non-identified proteins.

**Supplementary Table S9.** Summary of 20 experimentally identified proteins in *T. immobilis* containing a *Planctomycetes* specific cell surface motif.

**Supplementary Table S10.** Summary of 89 proteins of the *T. immobilis* subcellular proteome, which have been previously identified in cell wall preparations of this species (Mahajan et al. 2020a). Proteome Discoverer Scores from Mahajan et al. 2020a are listed as “Score.MS-19-041-1” and “Score-MS-19-041.2”.

**Supplementary Table S11.** Signal peptide predictions using SignalP-5.0 for homologous proteins to GMBLW1_25620 (WP_162656607.1).

**Supplementary Table S12.** Summary of unique proteins in membrane fractions of *G. obscuriglobus* (Sagulenko et al. 2017) and numbers of corresponding homologs in subcellular proteome of *T. immobilis*.

**Supplementary Table S13.** Summary of 369 *T. immobilis* homologs to *G. obscuriglobus*, which have been identified in isolated membrane preparations in Sagulenko et al. 2017. The *G. obscuriglobus* gene IDs and presence (“TRUE”)/absence (“FALSE”) in the respective membrane fraction suggested in Sagulenko et al. 2017 is indicated (gobs2: paryphoplasmic vesicles, gobs3: pore-containing membrane, gobs6: cytoplasmic membrane).

**Supplementary Table S14.** Summary of 524 experimentally identified proteins related to the COG categories J (Translation), K (Transcription), L (Replication and repair) and T (Signal Transduction) in *T. immobilis* and *E. coli*.

**Supplementary Table S15.** Summary of 173 experimentally identified proteins related to the COG categories J (Translation), K (Transcription), L (Replication and repair) and T (Signal Transduction) in *T. immobilis* and *E. coli*. Proteins were exclusively identified for one of three subcellular fractions: Tris soluble (S1), Tris/TX-100 soluble (S2) or Tris/SDS soluble (S3). BlastP searches (e-value cut-off 1e-10) were performed to identify *T. immobilis* homologs in *E. coli* and vice versa and corresponding hits are summarized in the column “blast_hits”.
